# Supplementary material for: Mitochondrial dynamics quantitatively revealed by STED nanoscopy with an enhanced squaraine variant probe
Source: Nat Commun. 2020 Jul 24;11:3699. doi: 10.1038/s41467-020-17546-1 (PMC7382495; doi:10.1038/s41467-020-17546-1)
Supplement: Supplementary file 1 — Supplementary Information [file 41467_2020_17546_MOESM1_ESM.pdf]

Supplementary Information

Mitochondrial dynamics quantitatively revealed by STED nanoscopy with an enhanced squaraine variant probe

Xusan Yang[1, Ɨ, *], Zhigang Yang[2, Ɨ, *], Zhaoyang Wu[1, Ɨ], Ying He[2], Chunyan Shan[3,4], Peiyuan Chai[3], Chenshuo Ma[5], Mi Tian[2], Junlin Teng[3], Dayong Jin[6], Wei Yan[2], Pintu Das[2], Junle Qu[2, *] and Peng Xi[1,6 *]

[1] Department of Biomedical Engineering, College of Engineering, Peking University, Beijing, 100871, China
[2] Key Laboratory of Optoelectronic Devices and Systems of Ministry of Education and Guangdong Province, College of Physics and Optoelectronic Engineering, Shenzhen University, Shenzhen, 518060, China
[3] School of Life Sciences, Peking University, Beijing, 100871, China
[4] National Center for Protein Sciences, Peking University, Beijing, 100871, China
[5] Material Science and Engineering, Rutgers University, Piscataway, NJ, 08854, United States
[6] UTS-SUStech Joint Research Centre for Biomedical Materials & Devices, Department of Biomedical Engineering, Southern University of Science and Technology, Shenzhen, China

[*]Email: xipeng@pku.edu.cn, jlqu@szu.edu.cn, zhgyang@szu.edu.cn, xy389@cornell.edu
Current address of Xusan Yang: School of Applied and Engineering Physics, Cornell University, Ithaca, 14853, United States
[Ɨ] These authors contributed equally to this work

Supplementary Figures

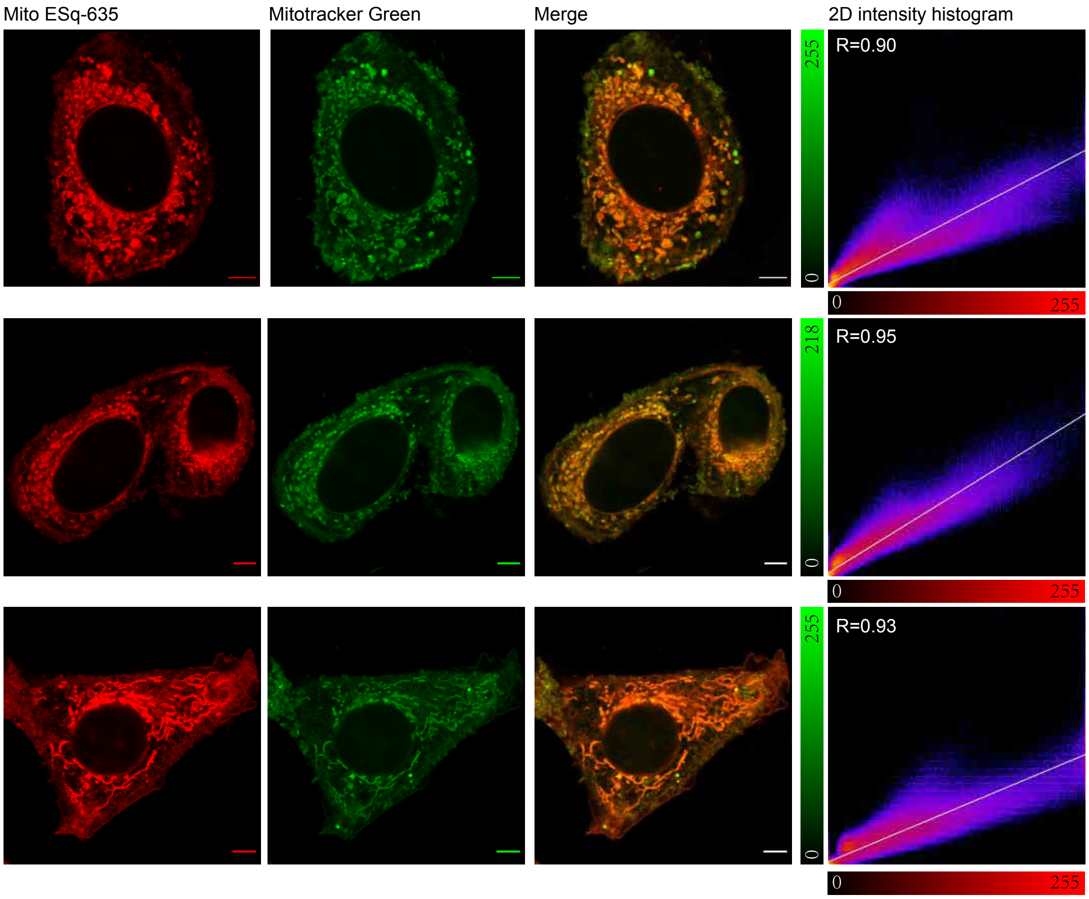

Supplementary Figure 1

Co-localization experiment in U2OS cells employing MitoTracker Green as golden standard for mitochondrial marker; the first column, enhanced squaraine dye (20 nM) labeled U2OS cells; the second column, MitoTracker Green(50 nM) labeled U2OS cells; the third column, merged images; the fourth column, 2D intensity histogram and Pearson's coefficients; excitation wavelength: MitoESq-635 (640 nm), MitoTracker Green (490 nm); detect range: MitoESq-635 (660-740 nm), MitoTracker Green (495-575 nm); Scale bar, 5 μm.

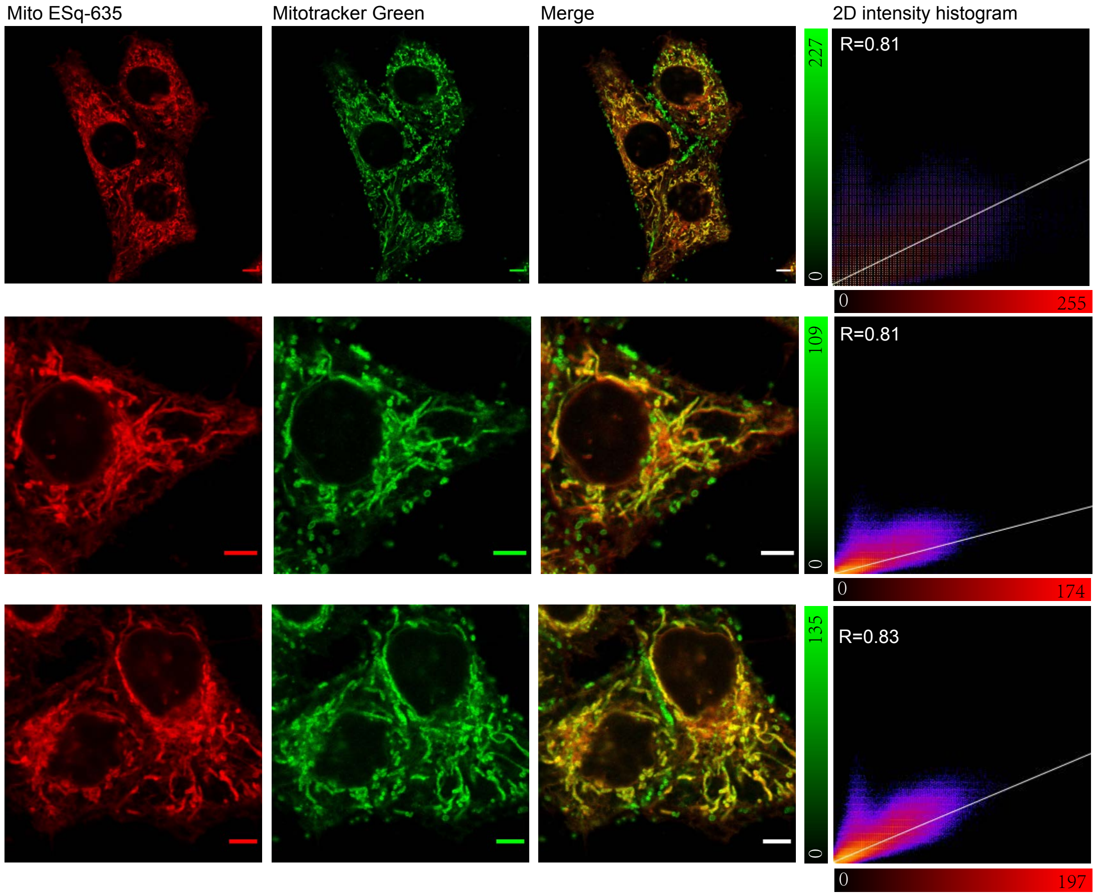

Supplementary Figure 2

Co-localization experiment in HeLa cells employing MitoTracker Green as golden standard for mitochondrial marker; the first column, enhanced squaraine dye (20 nM) labeled HeLa cells; the second column, MitoTracker Green (50 nM) labeled HeLa cells; the third column, merged images; the fourth column, 2D intensity histogram and Pearson's coefficients; excitation wavelength: MitoESq-635 (640 nm), MitoTracker Green (490 nm); detect range: MitoESq-635 (660-740 nm), MitoTracker Green (495-575 nm); Scale bar, 5 μm.

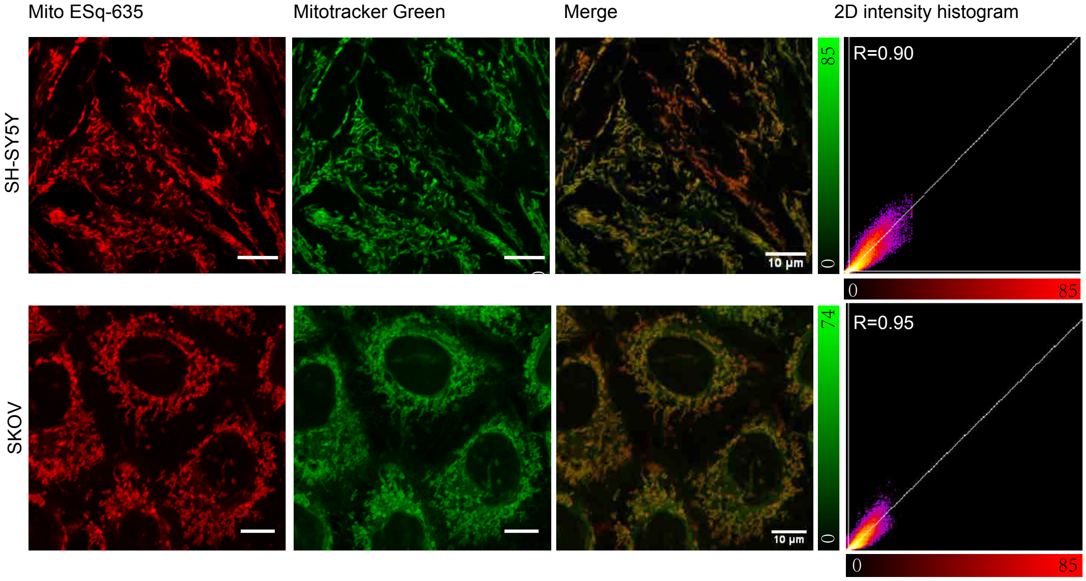

Supplementary Figure 3

Co-localization experiment in SH-SY5Y and SKOV cells employing MitoTracker Green as golden standard for mitochondrial marker; the first column, enhanced squaraine dye (20 nM) labeled SH-SY5Y and SKOV cells; the second column, MitoTracker Green(50 nM) labeledSH-SY5Y and SKOV cells; the third column, merged images; the fourth column, 2D intensity histogram and Pearson's coefficients; excitation wavelength: MitoESq-635 (640 nm), MitoTracker Green (488 nm); detect range: MitoESq-635 (700/75 nm), MitoTracker Green (525/50 nm); Scale bar, 10 μm.

From Supplementary Figure 1, 2, 3, it can be demonstrated that the probe (MitoESq-635) can be preferentially target for mitochondria in live cells.

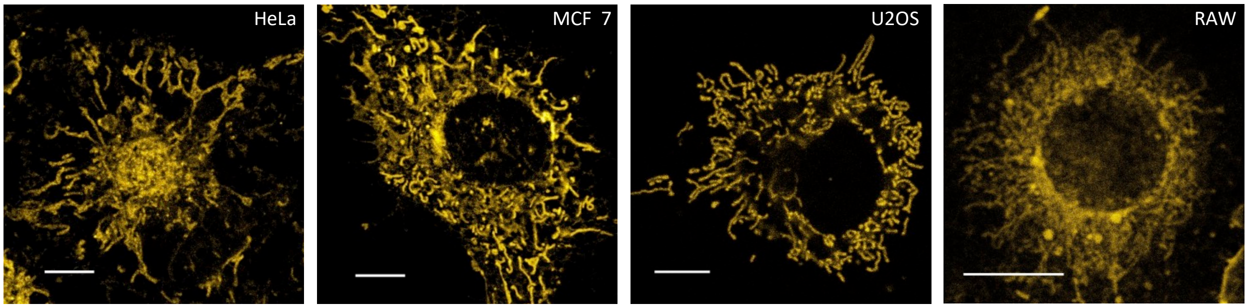

Supplementary Figure 4

Fluorescent imaging of MitoESq-635 in different cell lines (HeLa, MCF7, U2OS, and Raw264.7 cell). Scale bar, 10 μm.

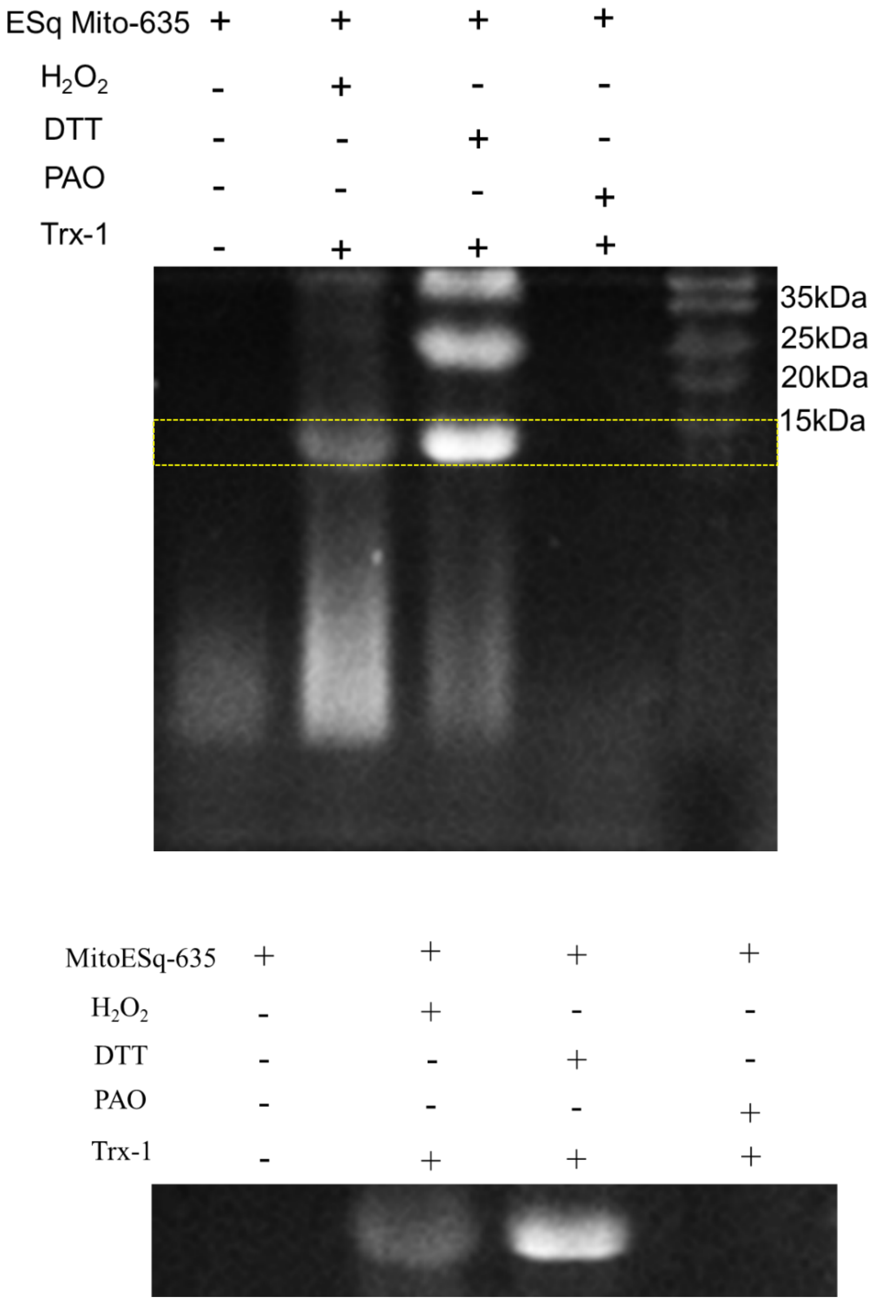

Supplementary Figure 5

SDS-PAGE identification of the covalent binding of MitoESq-635 to Thioredoxin proteins under different conditions, (upper figure, complete SDS-PAGE image; Lower figure, magnified image of SDS-PAGE results of ESq Mito-635 binding to Trx in the yellow box of upper figure); H_2O_2 (10 mM), DTT (10 mM), PAO (10 mM) were added into Thioredoxin protein (20 μM) solution separately, and incubated for 3 hours at 4 C°. After incubation, each sample was labeled with probe (50 μM) at 37 C° for 40 minutes. From the SDS-PAGE results, it can be seen that MitoESq-635 only in the presence of reduced Thioredoxin prepared by DTT, the probe can be covalently bound to the vicinal dithiols in the protein. In the presence of PAO, the vicinal dithiols on the Thioredoxin proteins were masked by PAO to inhibit the reactions of Thioredoxin with MitoESq-635. When H_2O_2 was added into above solution, the vicinal dithiols were oxidized into disulfide residue in the protein which disables the labeling reaction of MitoESq-635 with Thioredoxin. Therefore, DTT reduced the Thioredoxin (with vicinal dithiols) to promote the labeling of MitoESq-635 to mitochondria.

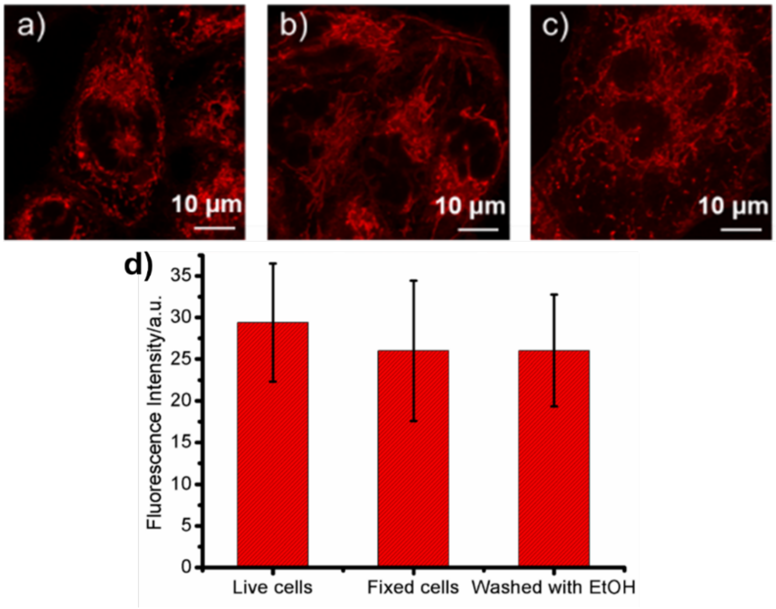

Supplementary Figure 6

Consistent with covalent binding of MitoESq-635 to the VDPs inside cells; confocal images of Hela cells stained with MitoESq-635 (0.5 μM) before (live cells) (a) and after cell fixation, (fixed cells). (b) After fixation, cells were incubated with pure ethanol (EtOH) for 5 minutes (washed out the free probe), then rinsed by PBS for three times (c) (d) Quantitative measurement of fluorescence intensities in (a), (b) and (c). Data are presented as the mean ±SD (n = 5). Compared with live HeLa cells, the probe was mainly localized in mitochondria with rare dispersion in the fixed HeLa cells, and even washed with pure ethanol to remove the free probe molecules. It is demonstrated that the probe molecules linked with the VDPs in mitochondria through phenarsenicate moiety very steadily.

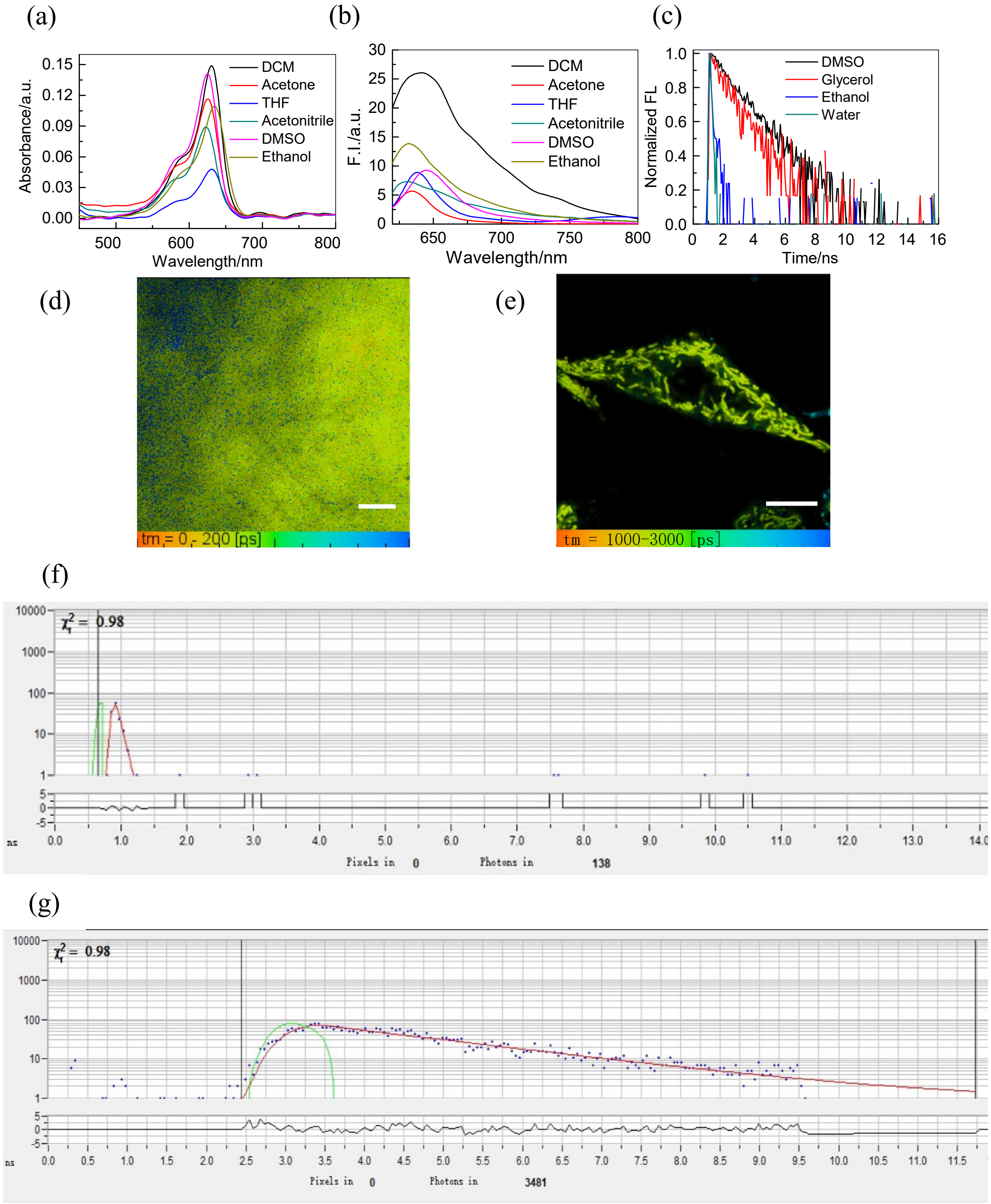

Supplementary Figure 7

Optical properties of MitoESq-635 in different solvents and living cells

(a-b) Absorption and Fluorescence spectra of MitoESq-635 in different solvents (c) Fluorescence lifetime decay curves of MitoESq-635 in different solvents (d-g) FLIM imaging of MitoESq-635 in HeLa cell and PBS which was measured by DCS-120 time-correlated single photon counting equipment (DCS-120, Becker Hickl), excitation

wavelength: 635 nm; detect range: 675/30 nm. On the mitochondria of Hela cells, the lifetime T≈1.7 ns. Meanwhile, the lifetime of MitoESq-635 in PBS (1 μM) is T_1≈65 ps. Scale bars for (d), (e) are 50 μm, and 10 μm respectively.

The relative fluorescence quantum yields were measured on a Horiba fluorescent spectrometer. And the fluorescence lifetime was measured on a commercially available equipment (DCS-120 time-correlated single photon counting equipment). Note that the fluorescence lifetime of MitoESq-635 is largely dependent on the environmental polarity. From the fluorescence decay curves in different solvents (e.g. DMSO, ethanol, glycerol, water, PBS), the lifetime in DMSO or glycerol was calculated to be 1.65, 1.69 ns, but cannot be detected in very polar solvents like ethanol or water in supplementary Figure 7c. And fluorescence lifetime was further measured in PBS and living cells. From the results in supplementary figures 7d and f, 7e and g, it can be found the lifetime was markedly increased in living cells (1.7 ns), due to the large changes of microenvironment.

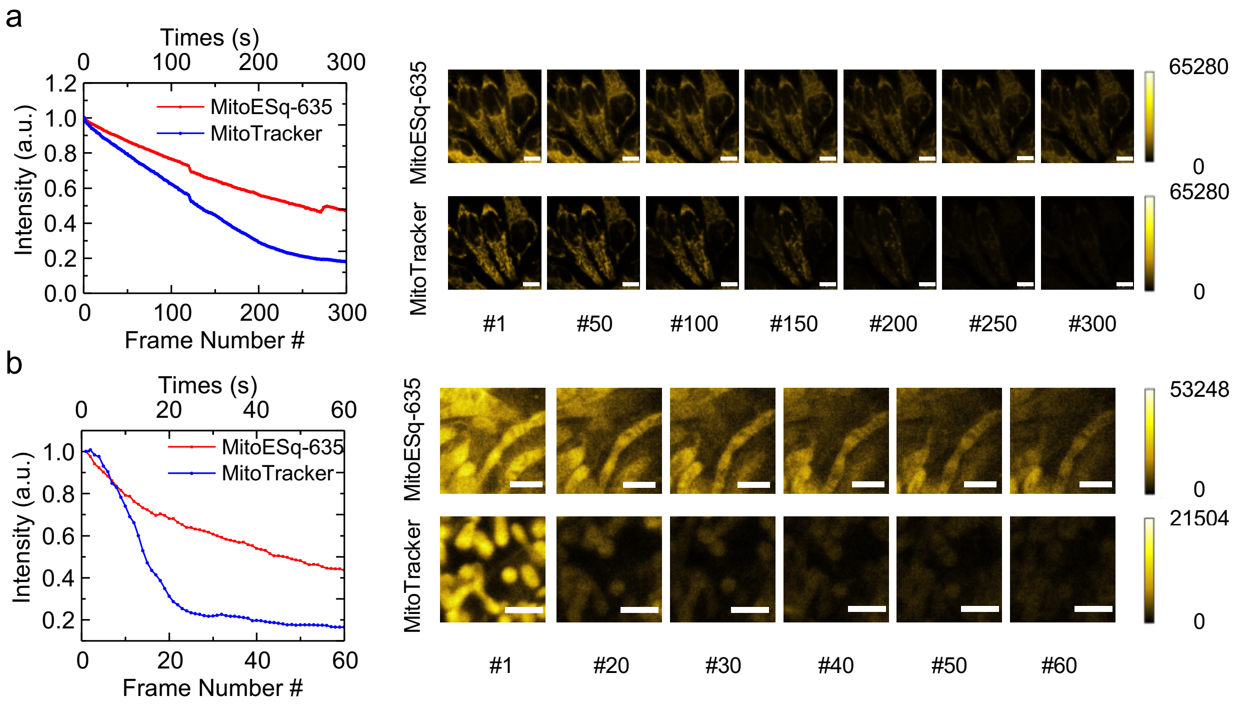

Supplementary Figure 8

Comparison of the photostability with MitoTracker and MitoESq-635.

(a) Comparison of the photostability under a confocal laser microscope of HeLa cells co-stained with 0.1 μM MitoESq-635 and 0.1 μM MitoTracker Green (Rhodamine123). Upper row, MitoESq-635, excited at 633 nm, and collected at 645-680 nm. Lower row, MitoTracker Green (Rhodamine123), excited at 488 nm, and collected at 500-560 nm. Confocal images are under the same imaging conditions, excitation under 1.97 μW averaged power, 1 frame per second acquisition speed (imaging time: 0.66s, recovery time: 0.34s). Fluorescence signal of each image are plotted as a function of the recorded image number. Scale bar, 10 μm. (b) Comparison of the photostability under a STED nanoscope of living HeLa cells stained with MitoESq-635 (0.1 μM) and MitoTracker DeepRed (0.1 μM), respectively. STED images are under the same imaging conditions, excitation under 1.1 μW averaged power at 640 nm, STED beam of 36 mW average power at 775 nm, 1 frame per second acquisition speed (imaging time: 0.66s, recovery time: 0.34s). Fluorescence signal of each image are plotted as a function of the recorded image number. Scale bar, 2 μm.

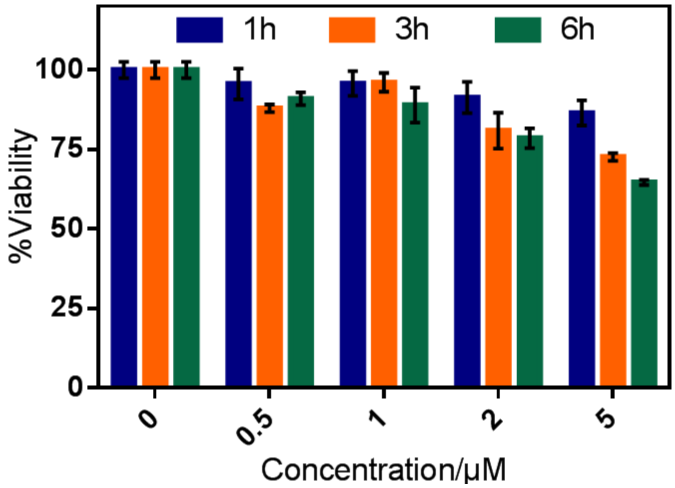

Supplementary Figure 9

Viability experiment on HeLa cells when incubating with MitoESq-635.

HeLa Cells were cultured with the probe with different concentrations (0, 0.5, 1.0, 2.0, 5.0 μM) for different time periods (1, 3 and 6 hours). It can be found in the figure that the probe exhibited low toxicity to HeLa cells, because cell viability was more than 70%, even at the concentration of 5.0 μM for 6 hours' incubation. Data are presented as the mean ±SD (n = 5).

In this experiment, a commercial chemical reagent of CCK-8, being nonradioactive, allows sensitive colorimetric assays for the determination of the number of viable cells in cell proliferation and cytotoxicity assays. Herein, we use the products from Dojindo (CCK-8) to carry out the cytotoxic assay. The detailed experimental procedures are listed as follows: 100 μL of HeLa cell suspension (about 5000 cells/well) was firstly dispensed in a 96-well plate, and was pre-incubated for 24 hours in a humidified incubator (37°C, 95% humidity, 5% CO_2). Then 10 μL of various concentrations of MitoESq-635 (final concentrations are 0, 0.5, 1, 2, 5 μM) were added to the 96-well plate to be tested, which the 96-well plate was incubated for different time (1, 3, 6 hours) in the incubator. And then 10 μL of CCK-8 solution was carefully added to each well of the above plate to avoid introducing bubbles into the wells. The obtained 96-well plate was incubated for another 3 hours in the incubator. And finally, the absorbance of the samples at 450 nm were measured using a microplate reader (Rayto RT-6100, Shenzhen, China).

$$\text{Cell Viability} = [(As\text{-}Ab) / (Ac\text{-}Ab)] \times 100\%$$

where As is absorbance of experimental wells (containing medium, CCK-8, MitoESq-635); Ac represents the absorbance of control wells (containing medium, CCK-8); Ab stands for the absorbance of blank wells (containing CCK-8).

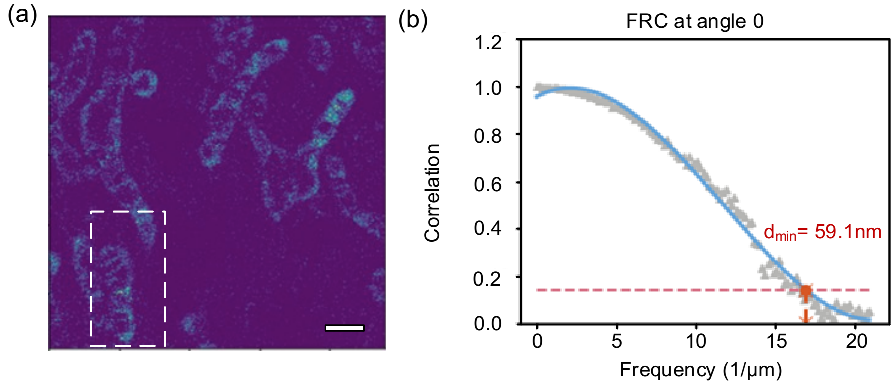

Supplementary Figure 10

FRC of Figure 2c. (a) White block shows Figure 2c image. Scale bar, 1 µm. (b) FRC shows the resolution of (a) is 59.1 nm, which is close to the resolution in Figure 2c (using intensity profile). The FRC is calculated by open software MIPLIB[1].

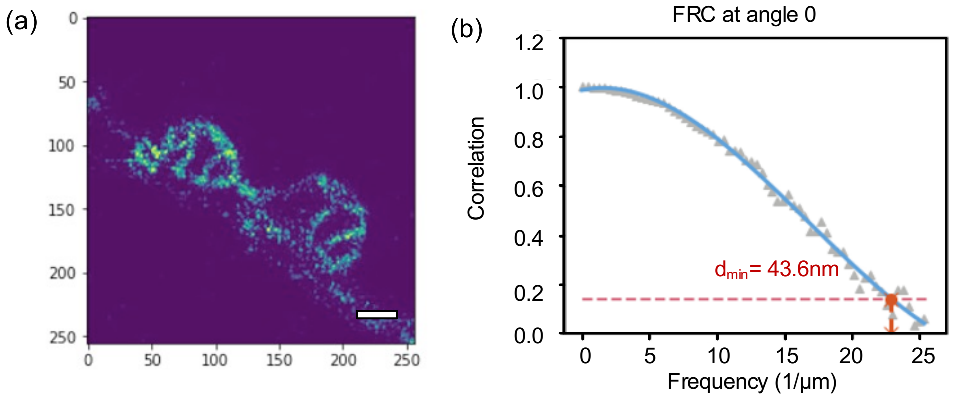

Supplementary Figure 11

FRC of Figure 4b. (a) Enlarge of figure 4b. 256×256 pixels, 16um per pixel. Scale bar, 0.5 µm. (b) FRC of (a) shows the resolution is ~ 43 nm. The FRC is calculated with open software MIPLIB[1].

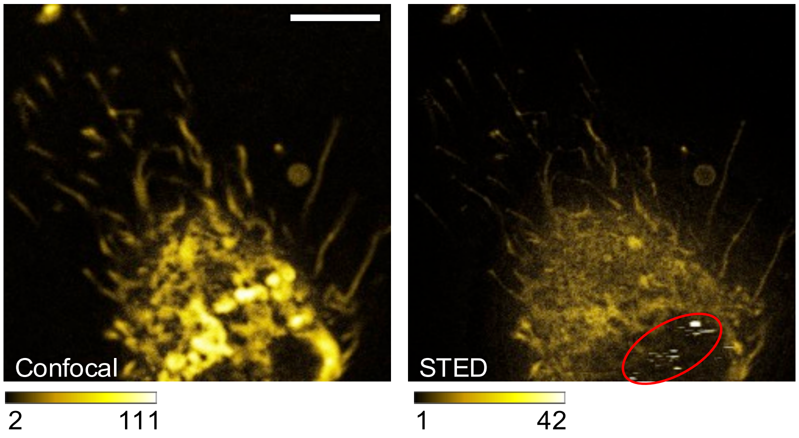

Supplementary Figure 12

Bursting of MitoESq-635 (0.5 µM) in HeLa cells caused by High dose depletion beam power in STED microscopy. The probe was subject to be incubated with HeLa cells for 30 minutes at 37 ºC before imaging. Depletion beam power, 51 mW. The molecules in red circle of STED image are bursting induced by high dose depletion beam, excitation wavelength: 633 nm, STED wavelength: 775 nm. Scale bar, 5 µm.

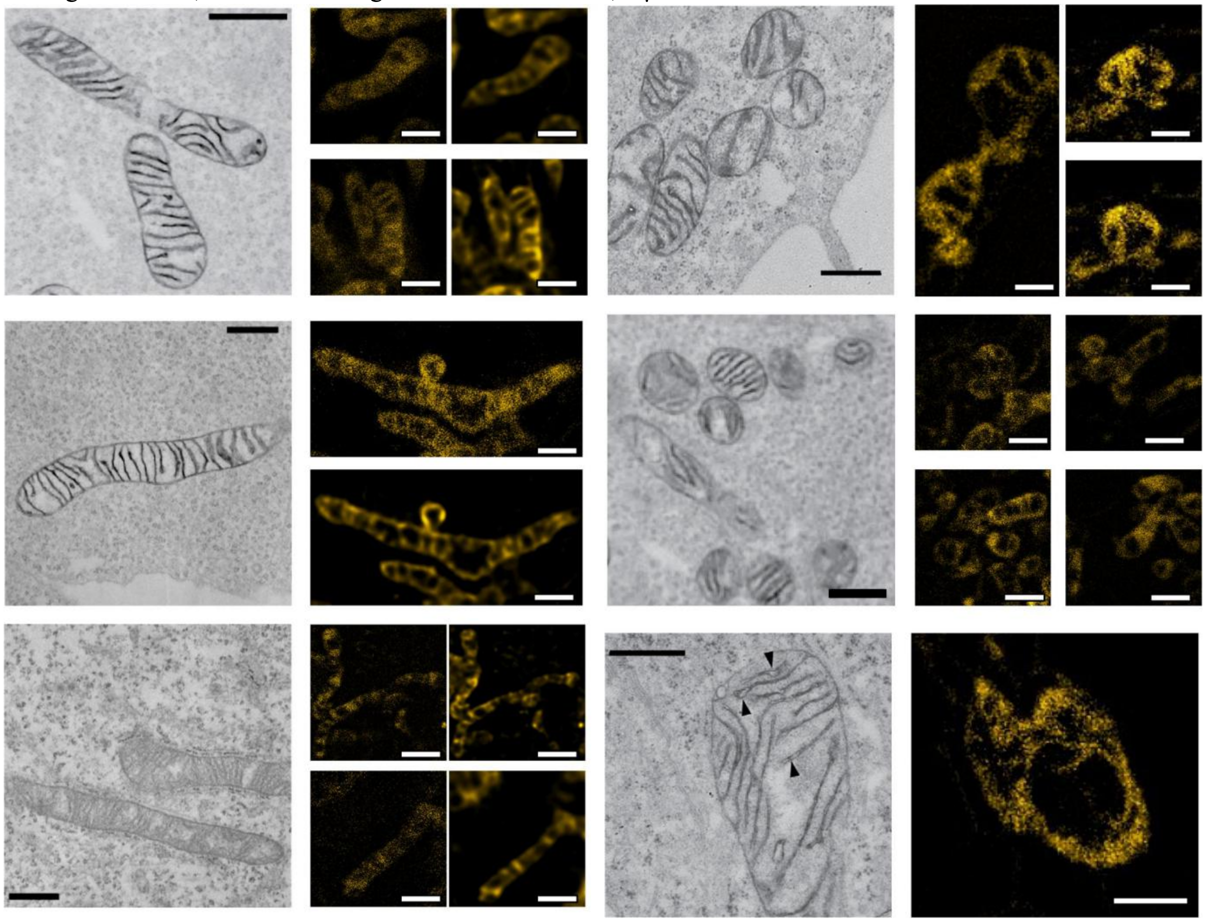

Supplementary Figure 13

The comparison of ultra-fine structure of mitochondria were resolved by STED and EM. Scale bar, black 500 nm, white, 1 µm. EM images were reprinted by permission from Springer Nature Customer Service Centre GmbH: Nature, Nature Methods (Directed evolution of APEX2 for electron microscopy and proximity labeling, Stephanie S Lam et al), Copyright (2014), (https://www.nature.com/articles/nmeth.3179)[2].

Supplementary Notes

Supplementary note 1

Synthesis procedures of compounds

Sample Preparation. All reagents including solvents and chemicals used were reagent grade. All reactions were performed under the argon atmosphere with dry, freshly distilled solvents under anhydrous conditions. Silica gel (100-200 mesh) was used for flash column chromatography for purifications. Water used in all experiments was doubly purified by Milli-Q System equipment (Double De-ioned-water). The solutions of compounds were typically prepared from 1.0 mM stock solutions in DMSO.

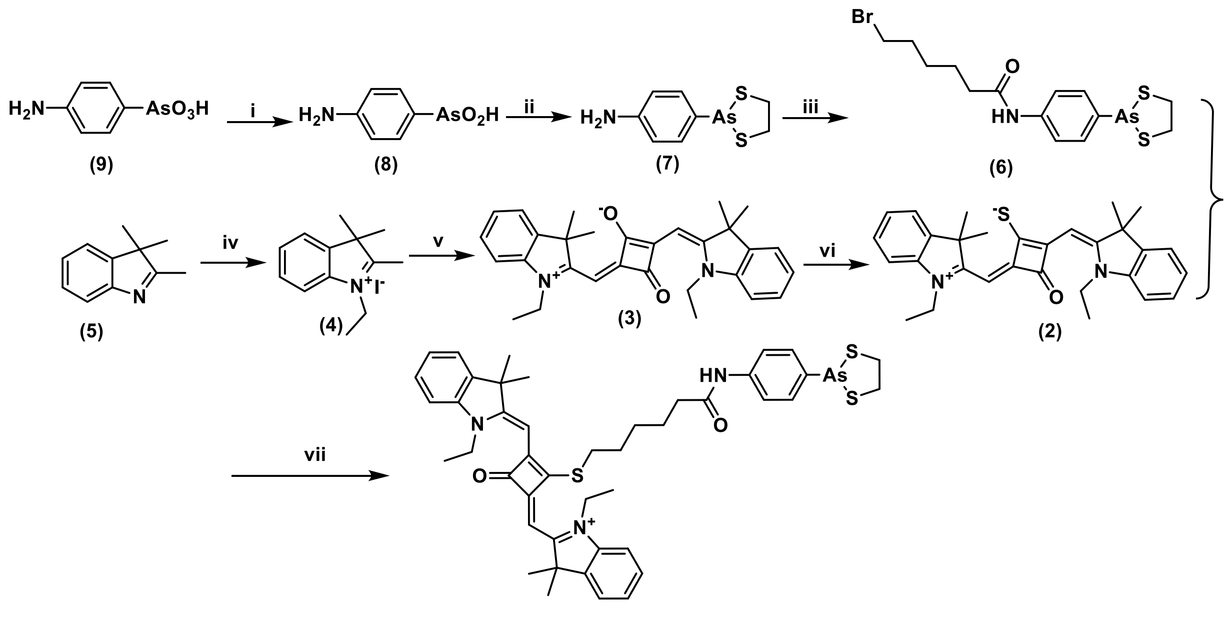

Supplementary Figure 14. Synthetic route of target compounds of squaraine derivative (MitoESq-635); i) phenylhydrazine, ethanol, reflux for 2h, yield 78%; ii) ethylenedithiol, ethanol, reflux for 0.5 h, yield 47%; iii) 6-bromohexanoic, HATU, DMAP, DIPEA, dry DMF, room temperature, stirred for overnight, yield 60%; iv) ethyl iodide, reflux 12 hs in toluene, yield 79.6%; v) squaric acid, triethyl orthoforamate, reflux for 8 hs in ethanol, yield 65%; vi) Lawesson's reagent, stirred for 10 hs in dry DCM/THF at 40 °C, 40%; vii) (**6**), NaI, stirred at 50 °C for 3 hs in anhydrous acetonitrile, 46%.

Synthesis of 4-aminophenylarsenoxide (8)

Compound **8** was prepared according to the literature procedure[3]. 4-aminophenylarsanilic acid **(9)** (11 g, 50 mmol) was dissolved in methanol (45 mL) and heated to reflux. Phenylhydrazine (12 mL, 110 mmol) was titrated dropwise to above mixture in 30 minutes. When N_2-production ceases, refluxing was continued for 2 hours. The mixture was condensed at 80 °C to exclude the solvent, then washed with water (50 mL) and an aqueous NaOH solution (0.2 M, 30 mL) and finally with diethyl ether (2 × 80 mL). The aqueous solution was treated with aqueous NH_4Cl solution (5 M, 40 mL) suspended overnight at 0 °C. Precipitates were collected through a Büchner funnel filter, washed by ice-water (20 mL× 2) and dried over KOH to give **8** as white powder (9.5 g, 78% yield).

Synthesis of 2-p-aminophenyl-1, 3, 2-dithiarsenolane (7) [3]

To a solution of **8** (2.6 g, 13 mmol) in dry ethanol (25 mL), ethanedithiol (1.5 mL, 20 mmol) was added dropwise and heated to reflux for 30 minutes with constant stirring. The solution was chilled in dry ice/acetone, co-distilled with toluene (106 mL) and concentrated. A white crystal was recrystallized from ethanol yielding 2.5 g (47% yields) of **9**. ^{1}H NMR (DMSO-d_6, 400 MHz): δ 3.13-3.36 (m, 4H), 5.40 (s, NH, 2H), 6.56 (d, J = 8.8 Hz, 2H), 7.27 (d, J = 4.8 Hz, 2H).

Synthesis of 4-(6-bromohexanoylamidophenyl)-1, 3, 2-dithiarsenolane (6)

In a 50 ml round bottom flask, 6-bromohexanoic acid (1.95 g, 10 mmol), HATU (2.5 g, 7 mmol), DMAP (0.12 g, 1 mmol) and DIPEA (0.5 mL) were added in dry DMF (15 mL) and the mixture was stirred for few minutes at room temperature. Then, 4-aminophenylarsenic **7** (1.3 g, 5 mmol) dissolved in DMF was added dropwise to above solution and stirring was continue overnight at room temperature. The reaction mixture was poured into saline solution (70 mL) and extracted with DCM (100 mL), dried over anhydrous Na_2SO_4. After evaporation of DCM, the product was purified by column chromatography to provide product **6** (2.6 g, yield 60%). ^{1}H NMR ($CDCl_3$, 400 MHz): δ 1.45 (m, J = 8.0 Hz, 2H), 1.72 (m, J = 8.0 Hz, 2H); 1.85 (m, J = 8.0 Hz, 2H); 2.37 (t, J = 8.0 Hz, 2H); 3.14 (m, J = 4.0 Hz, 2H); 3.33 (t, J = 8.0 Hz, 2H); 3.38 (t, J = 8.0 Hz, 2H); 7.53 (dd, J = 8.0 Hz, 4H); 8.06 (s, NH, 1H); ^{13}C NMR ($CDCl_3$, 100 MHz): δ 24.87, 27.93, 32.61, 37.51, 42.06, 119.95, 131.69, 138.80, 139.13, 171.79.

N-ethyl-2, 3, 3-trimethylindolinium iodide (4)

2, 3, 3-trimethyl-*3H*-indolenine (25 mL, 24.8 g, 156 mmol) and iodoethane (30 g, 192 mmol) were mixed in 50 mL dry toluene in 250 mL round flask, then the mixture was refluxed under argon atmosphere for 12 h, then stopped heating and cooled down. Then the precipitate was filtered

through a Buchner funnel, the solid product was washed by diethyl ether and dried in vacuum to afford pink product (39 g, yield: 79.6%), unnecessary to be further purified for the next reaction.

Synthesis of ethyl-squaraine dye (3)

Squaric acid (1.14 g, 10 mmol) was placed in a 250 mL round bottom flask equipped with a suitable magnetic stirring bar, then triethylorthoformate (2 mL) and dry ethanol (100 mL) were added into the flask; the obtained reaction mixture was heated to reflux under argon atmosphere and stirred till the white squaric acid completely dissolved and the solution became transparent; and then N-ethyl-2, 3, 3-trimethylindolinium iodide (**9**) (7g, 22 mmol) was added into the reaction mixture, continued to react under refluxing, the reaction process was monitored by TLC, the reaction was quenched until all the starting materials was consumed to give deep blue solution. The reaction was cooled down and solvent was removed under vacuum evaporation to give raw product in dark-blue solid. The squaraine dye (3) was purified by silica column chromatography with the gradient eluent of DCM/n-hexane in blue solid (**3**) (2.9 g, 65%). ^{1}H NMR (400 MHz, $CDCl_3$) δ 1.34 (t, J = 6.0 Hz, 6H); 1.73 (s, 12H); 4.04 (q, J = 6.0 Hz, 4H); 5.92 (s, 2H); 6.94 (d, J = 8.0 Hz, 2H); 7.08 (t, J = 8.0 Hz, 2H); 7.25 (t, J = 8.0 Hz, 2H); 7.30 (d, J = 8.0 Hz, 2H); ^{13}C NMR (100 MHz, $CDCl_3$) δ 21.91, 27.18, 38.52, 49.53, 85.92, 109.59, 122.39, 123.91, 124.31, 128.41, 142.43, 169.78, 179.51, 181.02, 182.26. HRMS (m/z): [M+H] + calcd. for $C_{30}H_{33}N_2O_2^+$, 453.2537; found, 453.2552.

Synthesis of sulfido-ethyl-squaraine dye (2)

The above prepared blue solid (**3**) (1.3 g, 3 mmol) was placed in a round bottom flask (150 mL) dissolved in dry THF/DCM and Lawsons's reagent (2.2 g, 5 mmol) was added into above solution to provide greenish reaction, which was stirred at 40 °C for 5 hours and monitored with TLC. The green spot was found to be on top of TLC plate with smaller polarity than dye (**3**). The reaction was cooled down to the room temperature. And then the solvent was removed by vacuum evaporation to give the residue with pungent smell, and the washed with saline water and the product was isolated *via* silica column chromatography to give sticky solid of (**2**), similar to a reported compound [4] (0.56 g, 40%), which was used for the next step without further purification. ^{1}H NMR (400 MHz, $CDCl_3$) δ 1.25 (s, 6H); 1.38 (t, J = 6.0 Hz, 6H); 1.79 (s, 6H); 4.05 (q, J = 6.0 Hz, 4H); 5.95 (s, 2H); 7.00 (d, J = 8.0 Hz, 2H); 7.15 (t, J = 8.0 Hz, 2H); 7.35 (t, J = 8.0 Hz, 2H); 7.58 (d, J = 8.0 Hz, 2H).

Synthesis of target compounds-As (MitoESq-635)

Sulfido-squaraine dye (2) (0.49 g, 1 mmol) was added into a round bottom flask (50 mL) in 20 mL dry acetonitrile, and ethylenedithiol-4-(6-bromohexanoylamido) phenylarsenicate (**6**) (0.18 g, 1.1 mmol) was added into above solution, which was heated at 50 ^{o}C for 3 hs under the protection of argon atmosphere monitored with TLC. Till complete disappearing of the starting materials, the reaction was cooled down to room temperature and the solvent was evaporated in vacuum. The residue was separated through silica column chromatography with the eluent of DCM/ methanol to give the blue solid (**1**) (0.38 g, 46% yield). TLC (CH_2Cl_2: MeOH, 95:5 v/v): R_f = 0.31 ^{1}H NMR (400 MHz, $CDCl_3$) δ 1.43 (t, J = 8.0 Hz, 6H), 1.70 (s, 12H), 1.83 (m, J = 6.0 Hz, 2H), 2.10 (m, J = 6.0 Hz, 2H), 2.72 (t, J = 6.0 Hz, 2H), 3.11 (m, J = 4.0 Hz, 2H), 3.28 (m, J = 4.0 Hz, 2H), 3.63 (t, J = 6.0 Hz, 2H), 4.21 (q, J = 8.0 Hz, 4H), 5.72 (s, 2H), 7.17 (d, J = 8.0 Hz, 2H), 7.27 (t, J = 8.0 Hz, 2H), 7.37 (d, J = 8.0 Hz, 2H), 7.46 (d, J = 8.0 Hz, 2H), 7.96 (d, J = 8.0 Hz, 2H), 9.69 (s, NH, 1H), ^{13}C NMR ($CDCl_3$, 100 MHz): δ 12.57, 25.19, 26.17, 27.59, 30.00, 32.27, 37.24, 40.74, 41.80, 50.64, 88.32, 111.49, 120.01, 122.58, 126.41, 128.87, 131.19, 137.11, 140.59, 141.01, 142.53, 171.82, 172.01, 173.08, 173.43, 174.15, 175.74; HRMS (m/z): $[M]^+$ calcd. For $C_{44}H_{51}AsN_3O_2S_3^+$, 824.2359; found, 824.2354.

Supplementary note 2

Possibility for wide applications in fluorescent labeling of enhanced squaraine dyes derivatives

In recent years, the traditional squaraine dyes (SQ) have attracted a lot of interests from the field of biological medical imaging.[5-7] In this work, we further developed a new squaraine dye with enhanced optical behaviors based on traditional SQ dyes which has great potential in biomedical imaging and diagnosis. To verify the wide applications in cellular imaging of the newly developed enhanced squaraine dyes (ESq), the sulfide-squaraine dyes (**2** in Supplementary Figure 14) were modified with different targeting ligand to carry out wide applications of STED imaging for biological targets in live cells. As demonstrated above, the alkyl sulfide-squaraine was suitable for STED imaging in live cells, as it was capable of STED imaging under low power of depletion laser and endurance of longtime laser illuminating, which revealed to be better than ATTO647N in STED imaging. The intracellular selective labeling of the probes mainly resorts to the targeting ligands, different targeting ligands conjugating with the sulfide-squaraine dye (**2**) will guide the obtained fluorescent probes to exactly mark the biological objects. As shown in the following figure, a new protocol was designed to prepare fluorescent probes for various objects. The intermediate sulfide-squaraine (**2)** was the first subject to reacting with haloalkyl acid to make a platform compound (**10)**, which can react with different targeting ligands with amines to provide target probes. The group R could be a lot of functional ligands for different objects inside cells, such as para-aminophenyl-arsenicate[8], phalloidine[9], glibenclamide[10], ceramide[11], indomethacin[12], and other antibodies or inhibitors *etc.*, to specifically label VDPs, cell backbone, ER, Golgi apparatus or single cyclooxygenase (COX) protein or other enzyme inside cells, respectively, which could achieve wide applications of the probes in live cell STED imaging strategies.

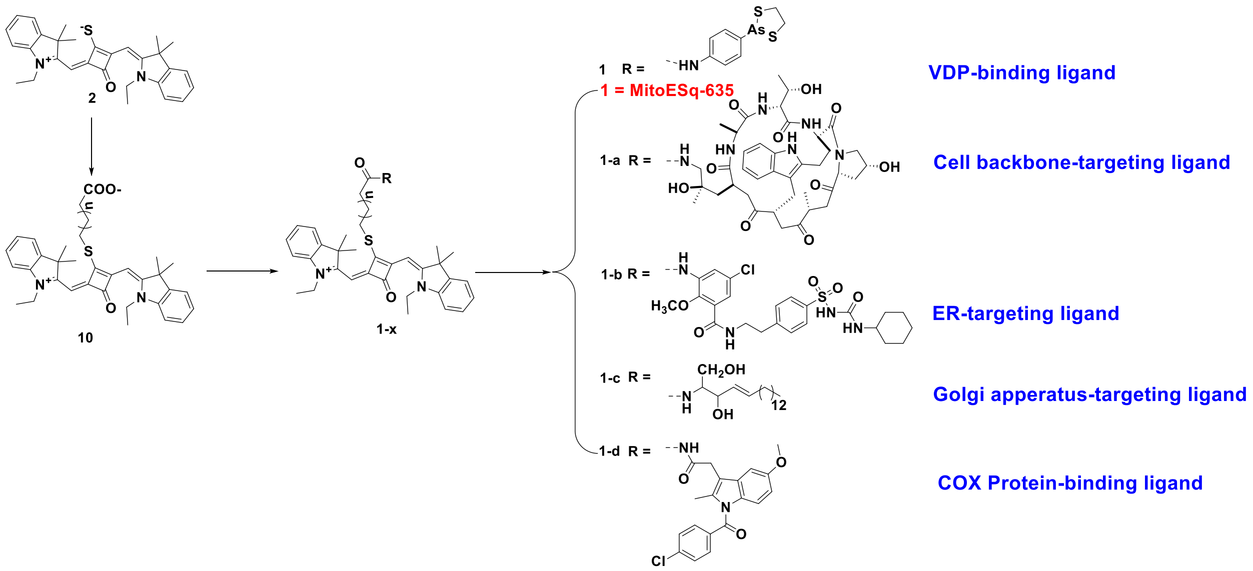

Supplementary Figure 15 Designing strategies for fluorescent probes for wide applications in STED imaging of live cells.

Supplementary note 3

MitoESq-635 covalently binding behavior to vicinal dithiols in mitochondria

As seen in Supplementary Figure 5, the probe was first incubated with commercial available VDP of thioredoxin, known as a kind of mitochondrial proteins containing a pair of dithiol residue in proximity on the surface of its reduced form, which can adjust mitochondrial redox homeostasis, under different conditions, the SDS-PAGE experimental results demonstrated that the probe can covalently attach to the protein in the presence of DTT. Moreover, MitoESq-635 can also bind to the proteins with vicinal dithiols in mitochondria of HeLa cells. The covalently binding behavior was verified *via* the comparison of fluorescence changes of MitoESq-635 in live HeLa cells, fixed cells and the washed fixed cells with pure ethanol, respectively (Supplementary Figure 6). Additionally, the colocalization experiment of MitoESq-635 with Rhodamine123 in HeLa cells were carried out to further identify the covalent binding of MitoESq-635 to mitochondrial VDPs As shown in Figure 1 d, in the live HeLa cells, the probe indicated well fluorescent overlapping with that of Rhodamine 123; whereas, when the cells were fixed with glutaraldehyde upon incubation with both probes for 30 minutes, MitoESq-635 was still kept in mitochondria without marked diffusion, not like Rhodamine 123 diffusing all over the cells and fluorescence decreasing markedly, which again substantiate the covalent binding of MitoESq-635 to VDPs in mitochondria.

Supplementary note 4

The relative fluorescence quantum yield and molar extinction coefficient measurement

Absorption and Fluorescence spectra were measured with a UV/Vis absorption spectrometer (GBC Cintra 2020, Australia) and a fluorescence spectrometer (Horiba iHR320, American), respectively. The relative fluorescence quantum yields were determined with Rhodamine B as a standard and calculated using the following equation (1):

$$\Phi_x = \Phi_s \frac{I_x A_s \lambda_{exs}}{I_s A_x \lambda_{exs}} \left(\frac{n_x}{n_s} \right)^2 \quad (1)$$

where A is absorbance at the excitation wavelength; I is the integration of the emission spectra; Φ_s represents the fluorescence quantum yield of the reference standard (Rhodamine B $\Phi_s = 0.97$ in ethanol solvents)[13]; λ_{ex} is the excitation wavelength; n is the refractive index of the solution (because of the low concentrations of the solutions (10^{-7}-10^{-8} mol/L), the refractive indices of the solutions were replaced with those of the solvents); and the subscripts x and s refer to the unknown and the standard, respectively.

Absorption coefficient:

$$\varepsilon = A / [c] \quad (2)$$

where A is absorbance at the excitation wavelength; [c] is the molar concentration. Here we have investigated the fluorescence quantum yields of MitoEsq-635 and fluorescence lifetime in solution and live ells, respectively. Firstly, we measured the relative fluorescence quantum yield and molar extinction coefficient of MitoESq-635 in different solvent (see Supplementary Table 1 and Supplementary Table 2).

Supplementary Table 1 Fluorescence Quantum yield of Mito-ESq-635 and its relative parameters

	λ_x	A_x	I_x	η_x	Φ_x
Ethanol	610	0.10	1046.68	1.10	0.18
DCM	610	0.13	1133.13	1.42	0.26
DMSO	610	0.11	835.29	1.48	0.25

λ_x the excitation wavelength; A_x the absorbance of MitoESq-635 at the excitation wavelength; I_x the integral of the emission spectral curve of MitoESq-635; η_x the refraction index of solvents; Φ_x fluorescence quantum yields of MitoESq-635

Supplementary Table 2 Molar Extinction coefficient of Mito-ESq-635

Solvents	DCM	DMSO	EtOH	MeOH	H_2O
$\varepsilon(\times 10^5/M^{-1}.cm^{-1})$	0.96	0.6	1.58	1.0	0.59

Supplementary note 5

Supplementary Table 3 Comparation of different results

Videos	Image time: t1 (s)	Rest time: t2 (s)	Total frame time: t3=t1+t2	Frames (F)	Video time: t4=t3*F (s)	Logical size	Pixel size (nm)	Excitation power	STED power (mW)	Resolution (nm)
SI Video 1a	2.58	0.42	3 s	200	600	128*128	22	5 µW	8.96	70
SI Video 1b	2.58	0.42	3 s	200	600	128*128	31	5 µW	7.84	80
SI Video 1c	2.58	0.42	3 s	200	600	128*128	23	2.5 µW	6.72	80
SI Video 4	2.58	0.42	3 s	60	180	512*512	28	5 µW	7.84	70
SI Video 6	2.58	0.42	3 s	120	360	400*128	24	5 µW	7.84	N/A
Scientific Report[14]	N/A	N/A	15 s	10-20	120	N/A	20-25	N/A	N/A	70
PNAS[15]	N/A	N/A	1.3 s	300	390	N/A	N/A	N/A	108	60
Hessian SIM[16]	N/A	N/A	5.3 ms 188 HZ	800	4.2	N/A	N/A	66.15 W/cm^2	N/A	90

N/A: Not applicable

Supplementary Table 4 Comparation of different probes

Probe	Molecule specificity	Wavelength	Frame time	Video time	Frames and time before swelling	Excitation power	STED power	Resolution	Data processing	Operational complexity
MitoESq-635	N/A	Excitation: 635 nm, STED: 775nm	2.58 s	600s, 200 frames	No swelling for < 100 frames, 300s	5 µW	7.84 mW	<50 nm	Raw data	One step
SNAP-tag[14]	COX8A-SNAP fusion proteins	Excitation: 640 nm, STED: 775nm	15 s	120s, 10-20 frames	10-20 frames, 120s	N/A	N/A	70 nm	Raw data	Complex
Mito PB Yellow[15]	N/A	Excitation: 488 nm, STED: 660nm	1.3 s	390s, 300 frame s	About 100 frames, 130s	N/A	108 mW	60 nm	Deconvolution	N/A

N/A: Not applicable

Note that the resolution in Figure 3a and Supplementary Video 4 are better than that of Hessian SIM, although SIM has a faster frame speed as shown in Supplementary Table 3. Our results have longer video time than that of supplementary reference[14] and need less STED power which means less photodamage than supplementary reference[15], although their resolutions are similar as shown in Supplementary Table 4.

Supplementary note 6

Phototoxicity and photodamage in super-resolution microscopy

The real promise of super-resolution is the ability and the hope of living cell dynamics imaging. But it's of big challenges to fulfill this promise. In order to map subcellular structure at nanoscale, much more photon should be attained for get a decent signal to background ratio. Cells have a limited tolerance for light intensity because of photodamage and photo-toxicity. However, these super-resolution methods including (d) STORM/PALM and STED microscopy require high doses of light to achieve resolution enhancement breaking diffraction limit.

Photo-toxicity depends on both light dose and wavelength, as well as the site environment and cell type of employed fluorophore. Generally, lower laser power and longer wavelength are decent strategies to avoid unwanted photo-toxicity. To get effective information by bioimaging, images with enough contrast, spatial, and temporal resolution should be attainable while leave the biology of interest intact. Contrast, spatial and temporal resolution are dependent t to each other. Usually, better contrast, higher spatial or temporal resolution requires much higher light (excitation or modulation) dose and/or more fluorescence photons, while intense light illumination will cause marked photo damage/toxicity in turn. Minimizing photo damage/ toxicity is the most key strategy to carry out living cell imaging because any imaging of biological dynamics/ process will come to the end once the photo-toxicity occurs in the samples. Therefore, the probe which can achieve enough contrast, spatial resolution, and temporal resolution with modest light dose (excitation or modulation) is highly desired. Considering the fluorescence photon budget for living cell imaging, luminescent probes should be excited under low power of light and have enough emission photons for a decent signal to noise ratio. MitoESq-635 has excellent photophysical property over golden standard organic dye ATTON 647 N probe for STED nanoscopic imaging because it has excitation and depletion spectrum in red and infrared as well as lower saturated intensity.[17]

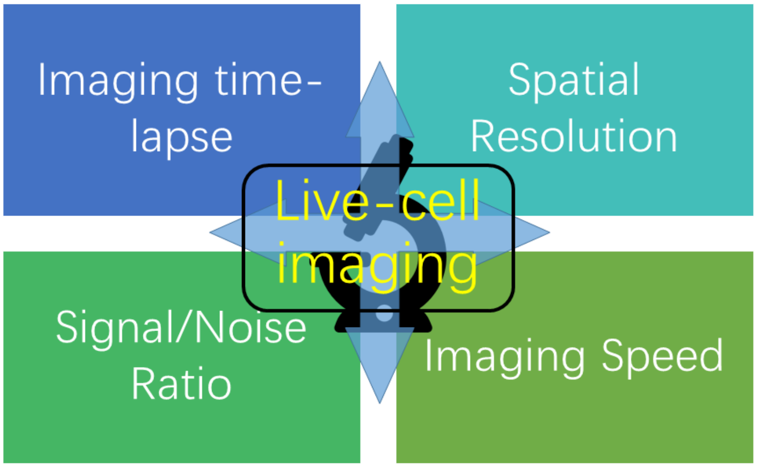

Supplementary Figure 16 The four main considerations for live imaging.

Photon-induced cell damage is thus a crucial consideration for light microscopy of living samples, and minimizing it is a fundamental concern. Many factors can influence phototoxicity including fluorophores (concentration and their subcellular localization), excitation wavelength and intensity (peak and time-averaged), exposure time (scanning time and the amount of dark recovery time between images), sample preparation, media, singlet oxygen concentration, cell type and age, the developmental stage of the organism, and synergistic effects of experimental perturbations, which can all affect a live sample under fluorescence microscopic observation.

It is a complex phenomenon consisting of wavelength-dependent photophysical mechanisms that can generate highly reactive photochemical products, heat, and DNA damage. The phototoxicity by the damaging radicals produced by laser excitation can be minimized, but unavoidable. Cells can tolerate laser illuminating as long as their defense mechanisms are not overwhelmed. During the fluorescent imaging, fluorescent proteins or dyes in their excited state are readily to generate reactive oxygen species (ROS). These unstable, short-lived reactive species may in turn damage the chemical structure and biological function of proximal biomolecules, so any type of fluorescence microscopy risks causing light-induced damage to living samples. The degree to which illumination causes photodamage depends strongly on the sample tolerance, the duration of the observation, and the observed cellular process.

Mitochondria are intricately involved in the activation and regulation of different pathways adjusting the balance between cell death and survivals. Complexes I and III in the inner mitochondrial membrane are the key producers of ROS that plays crucial roles in mitochondrial

biology. Mitochondrial membranes become depolarized at excessive ROS and may be destroyed in a ROS burst by the prolonged opening of mitochondrial permeability transition pores. This mitochondrial permeability transition can further induce DNA fragmentation and cell apoptosis. The mitochondrial membrane contains key regulators (cytochrome c, Bcl-2 family of proteins), which play vital roles in the homeostasis between apoptosis and survival. Even at low irradiance, mitochondria-derived ROS may lead to cell death. One possible reason is that mitochondria are rich in chromophores that are photosensitive in the UV and visible wavelength range, such as heme proteins, flavoproteins, and NAD(P)H. This is compounded by the complex interaction of mitochondria with the ER. Such interactions leading to cell death are exploited in photodynamic therapy, where selective photosensitizers are used to generate large amounts of ROS through illumination. This field is also a rich source for studies of localized ROS damage to cell populations, tissues, and organs. Although specimens show diverse signs of light-induced damage, common themes do exist. A frequent recommendation for assessing phototoxicity is to look for telltale morphological signs such as cellular swelling and rounding, blebbing, or the appearance of vacuoles.[18]

(III) Chemical Structure Identifications

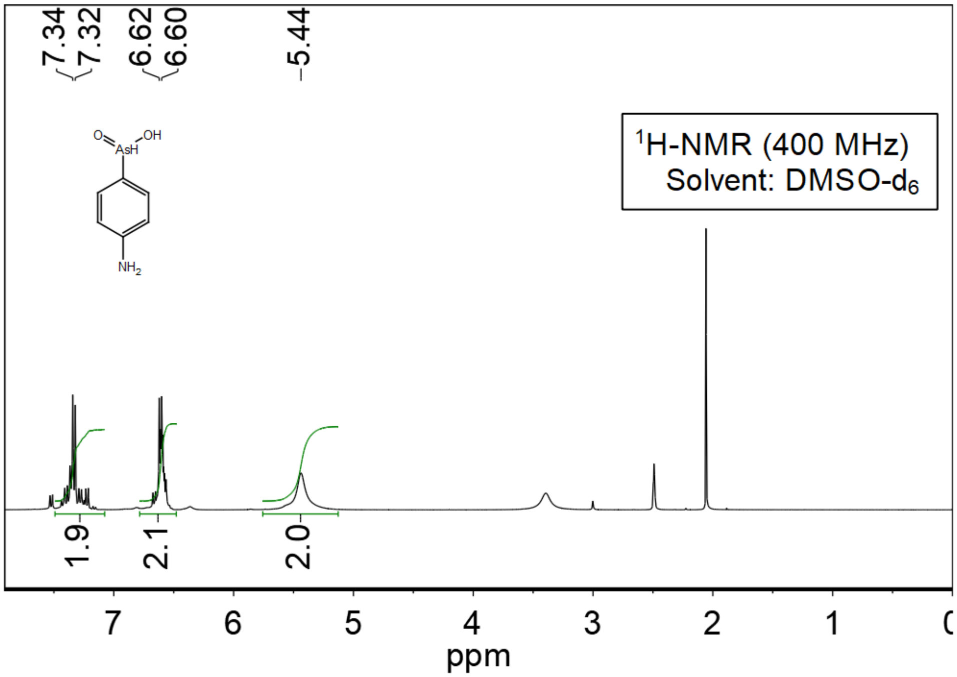

Supplementary Figure 17
Chemical Structure Identifications of compounds

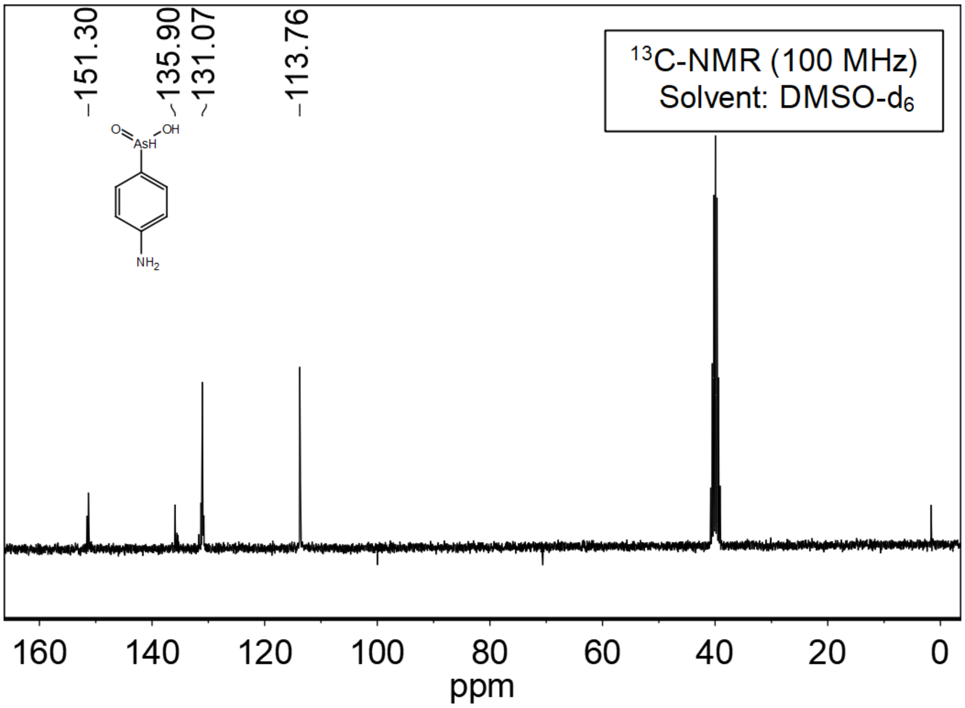

Supplementary Figure 18
^{1}H and ^{13}C NMR spectra of compound 8

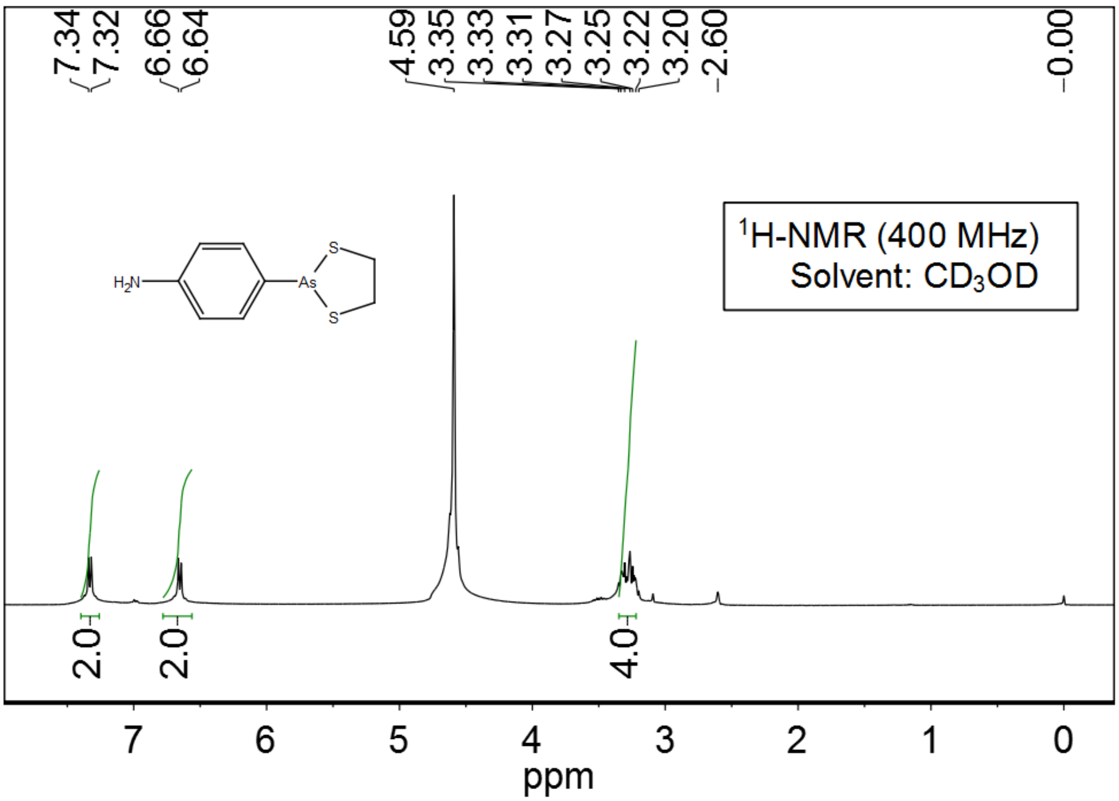

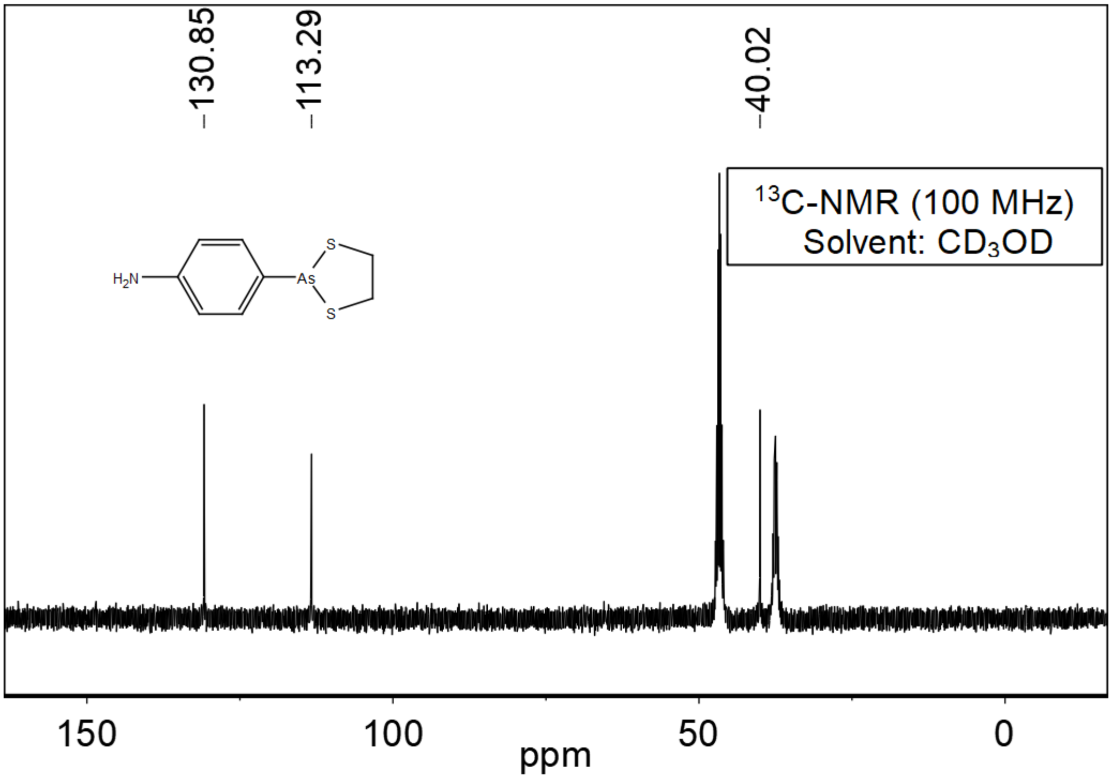

Supplementary Figure 19

^{1}H and ^{13}C NMR spectra of compound 7

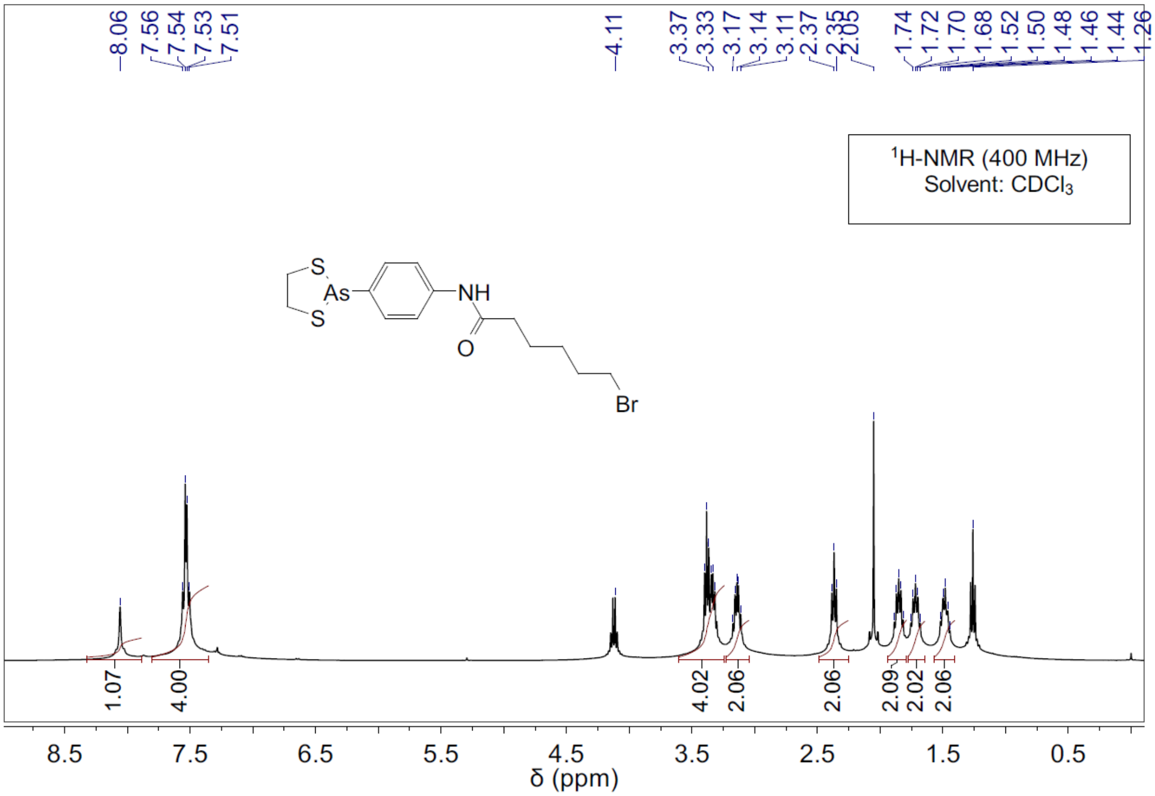

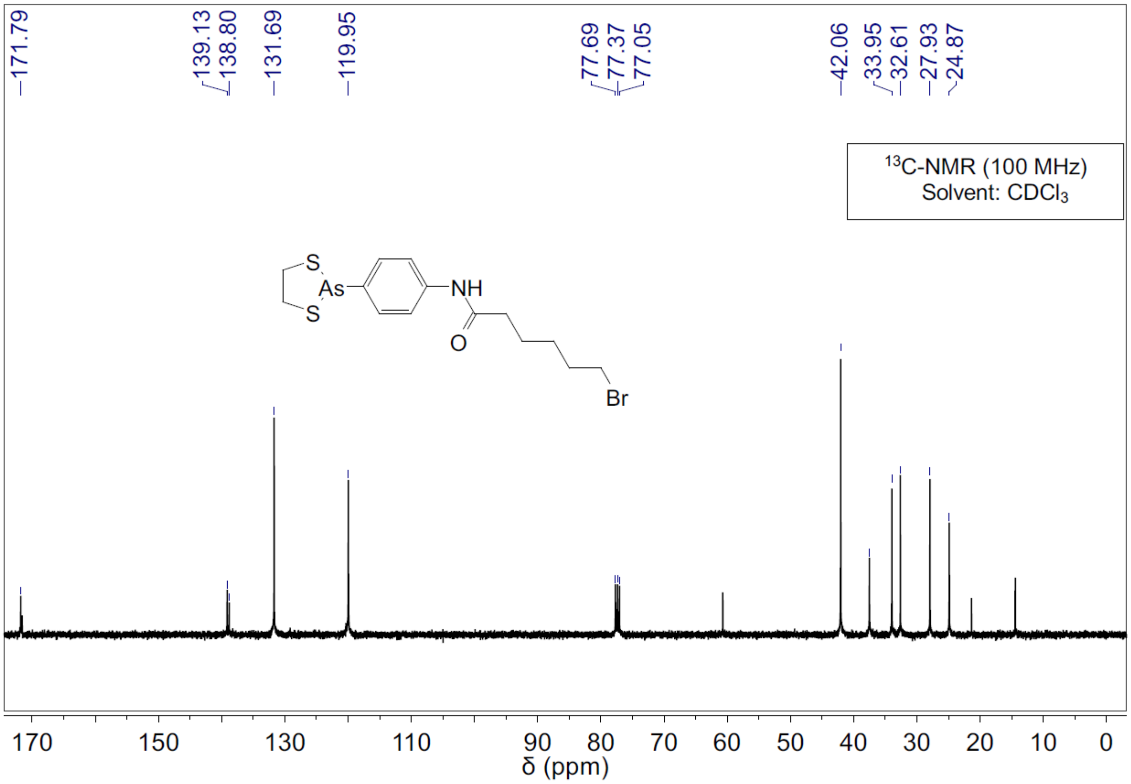

Supplementary Figure 20

^{1}H and ^{13}C NMR spectra of compound 6

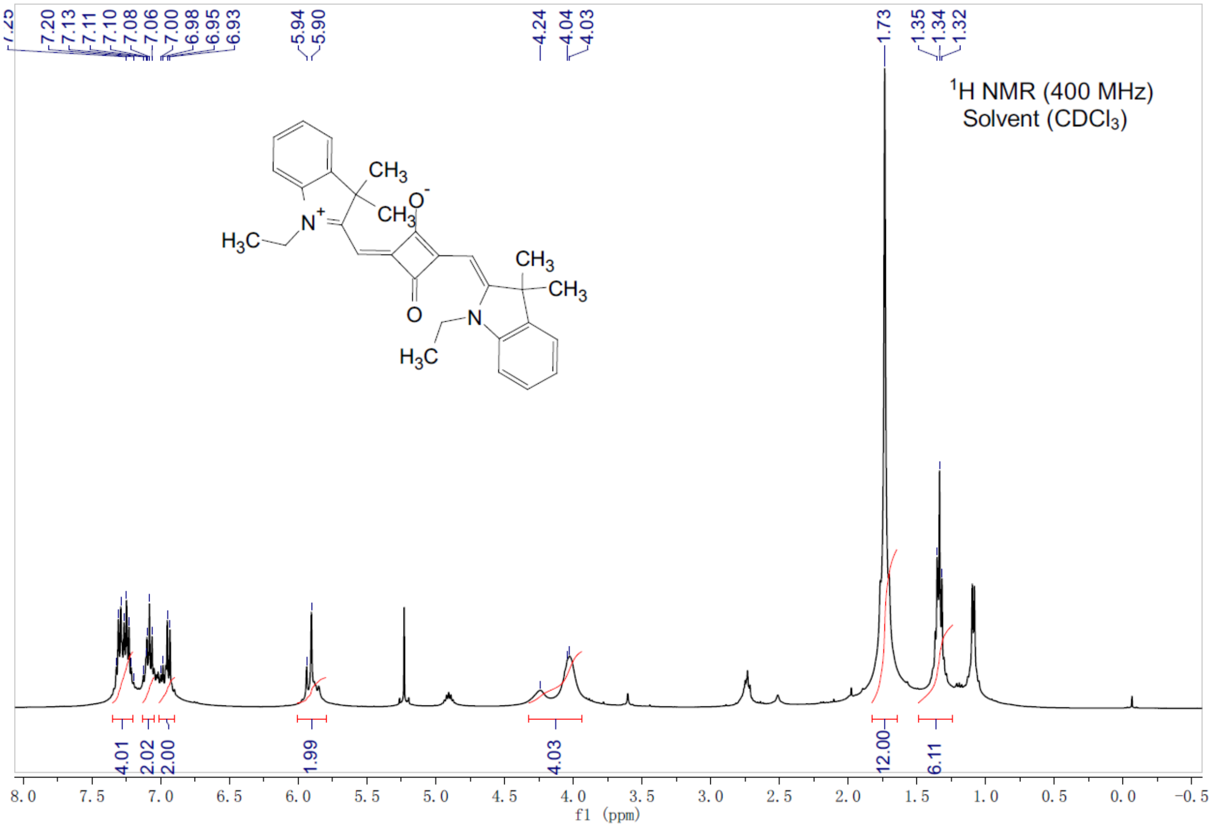

7.25
7.20
7.13
7.11
7.10
7.08
7.06
7.00
6.98
6.95
6.93
5.94
5.90
4.24
4.04
4.03
1.73
1.35
1.34
1.32
^{1}H NMR (400 MHz)
Solvent ($CDCl_3$)
CH3
O-
N+
CH3
H3C
CH3
CH3
O
N
H3C
4.01
2.02
2.00
1.99
4.03
12.00
6.11
f1 (ppm)

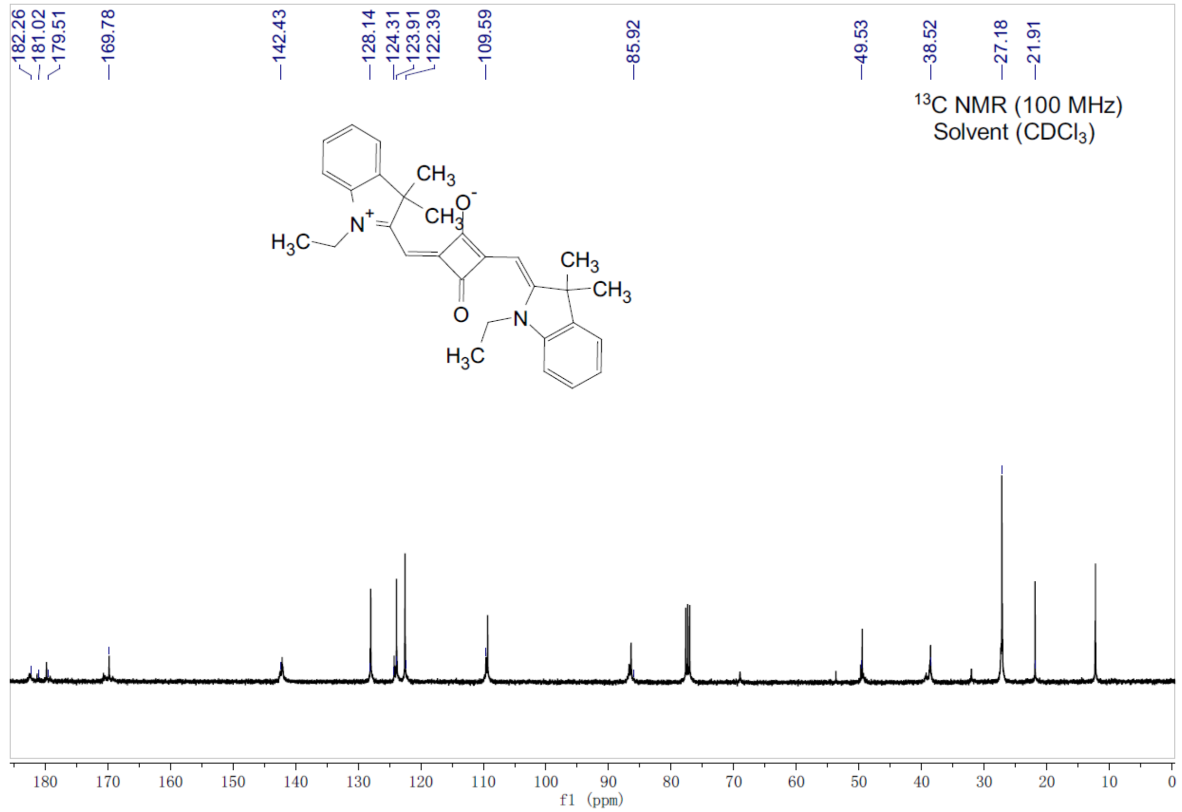

182.26
181.02
179.51
169.78
142.43
128.14
124.31
123.91
122.39
109.59
85.92
49.53
38.52
27.18
21.91
^{13}C NMR (100 MHz)
Solvent ($CDCl_3$)
CH3
O-
N+
CH3
H3C
CH3
CH3
O
N
H3C
f1 (ppm)

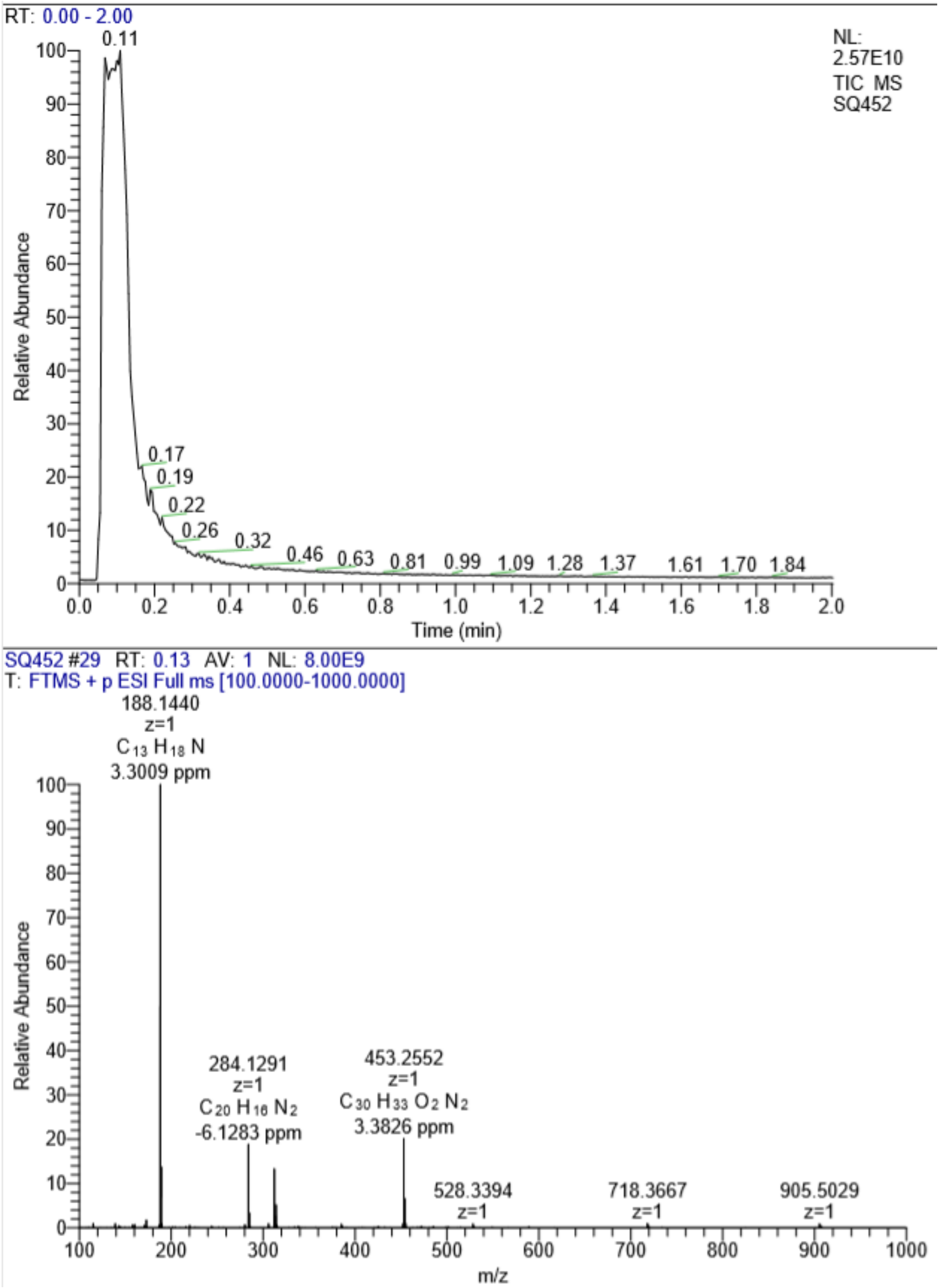

Supplementary Figure 21

^{1}H and ^{13}C NMR and High-resolution Mass Spectra of compound 3

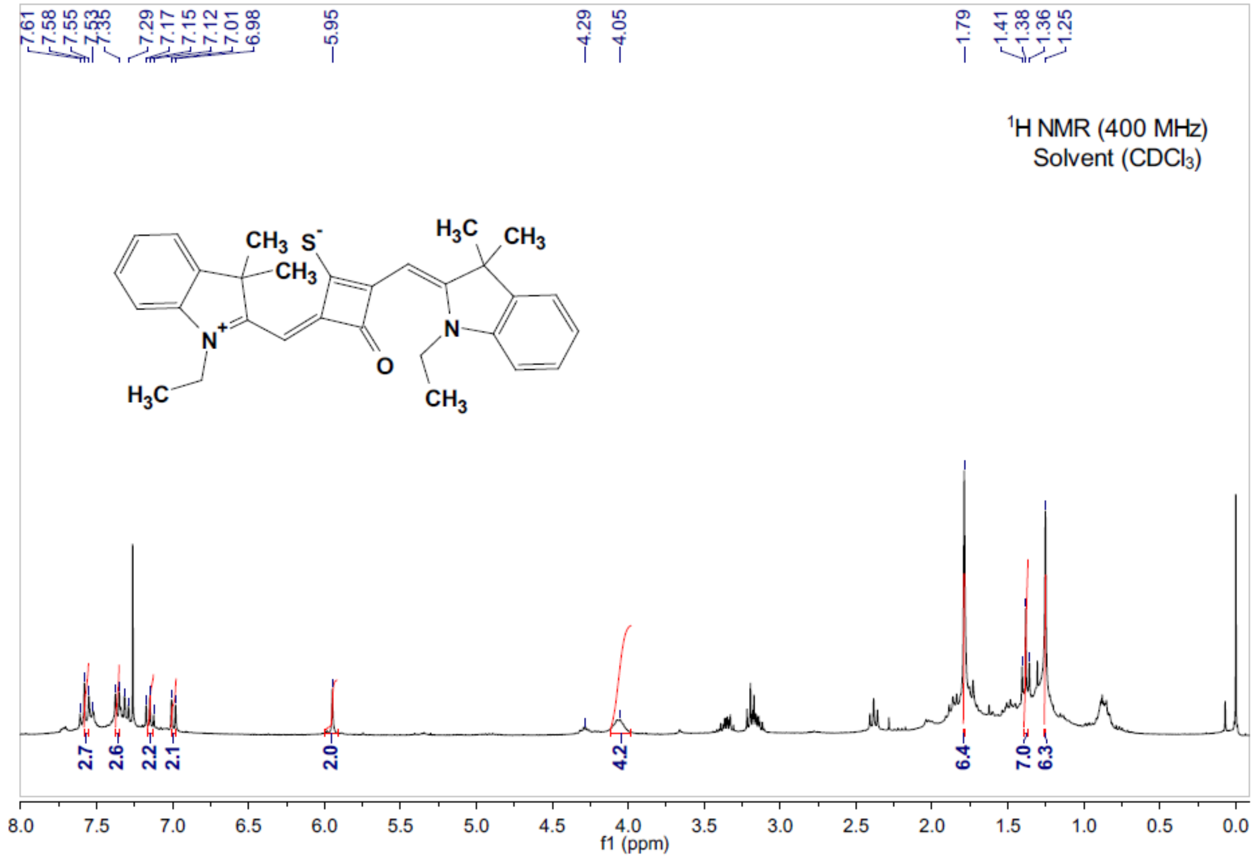

Supplementary Figure 22

^1H spectra of compound 2

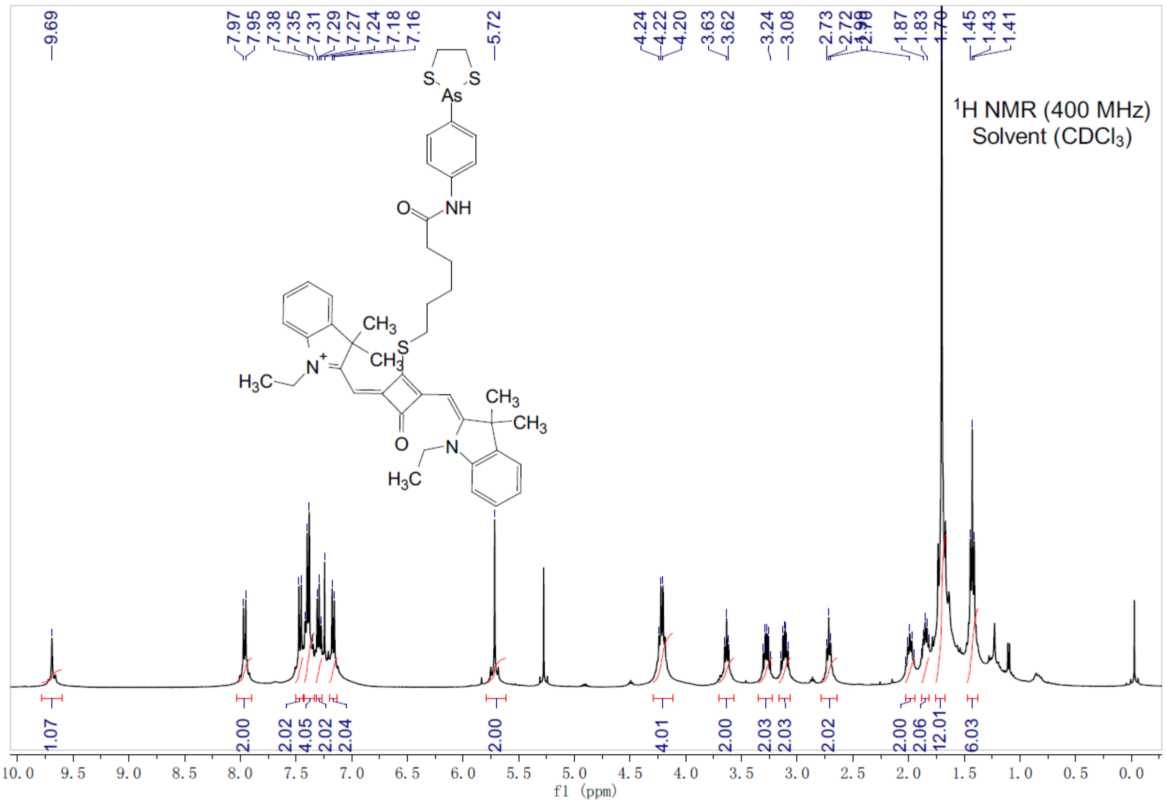

9.69
7.97
7.95
7.38
7.35
7.31
7.29
7.27
7.24
7.18
7.16
5.72
4.24
4.22
4.20
3.63
3.62
3.24
3.08
2.73
2.72
1.87
1.83
1.70
1.45
1.43
1.41
^{1}H NMR (400 MHz)
Solvent (CDCl$_3$)
1.07
2.00
2.02
4.05
2.02
2.04
2.00
4.01
2.00
2.03
2.03
2.02
2.00
2.06
12.01
6.03
f1 (ppm)

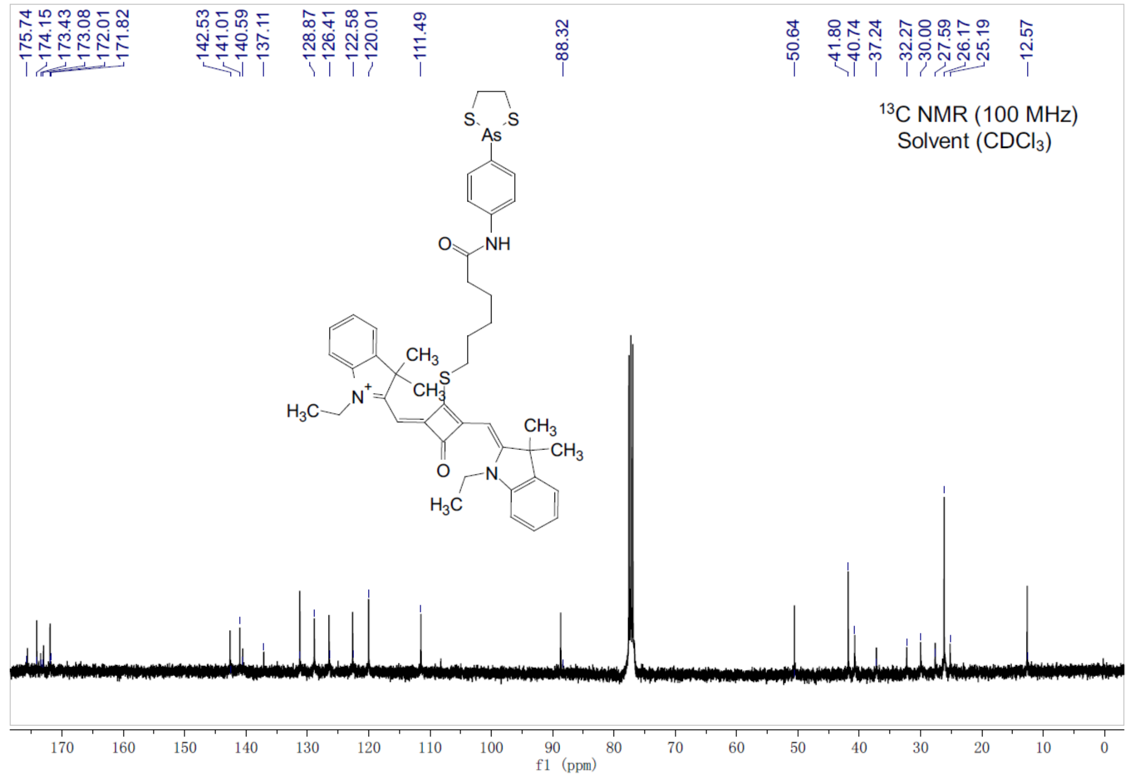

175.74
174.15
173.43
173.08
172.01
171.82
142.53
141.01
140.59
137.11
128.87
126.41
122.58
120.01
111.49
88.32
50.64
41.80
40.74
37.24
32.27
30.00
27.59
26.17
25.19
12.57
^{13}C NMR (100 MHz)
Solvent (CDCl$_3$)
f1 (ppm)

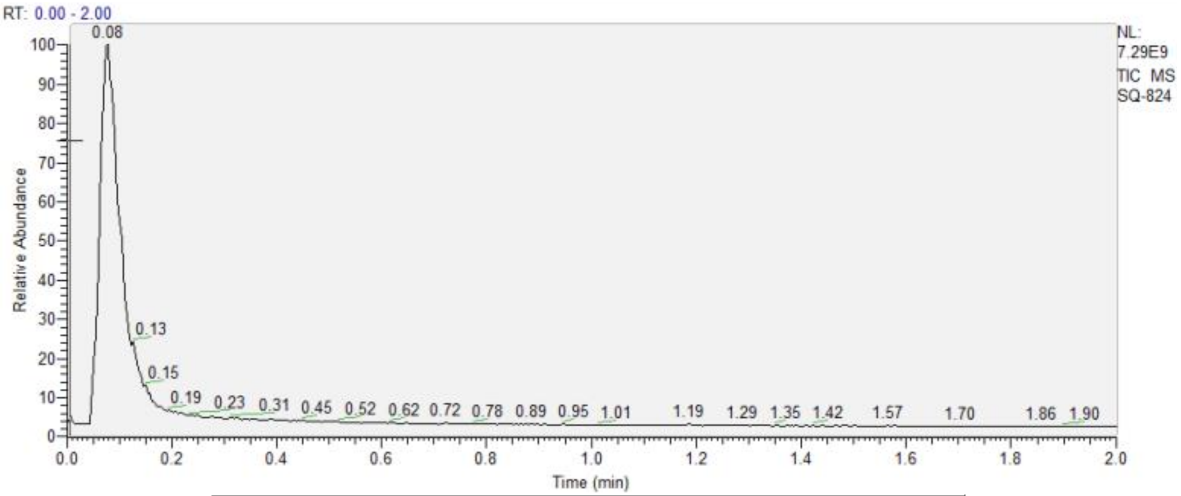

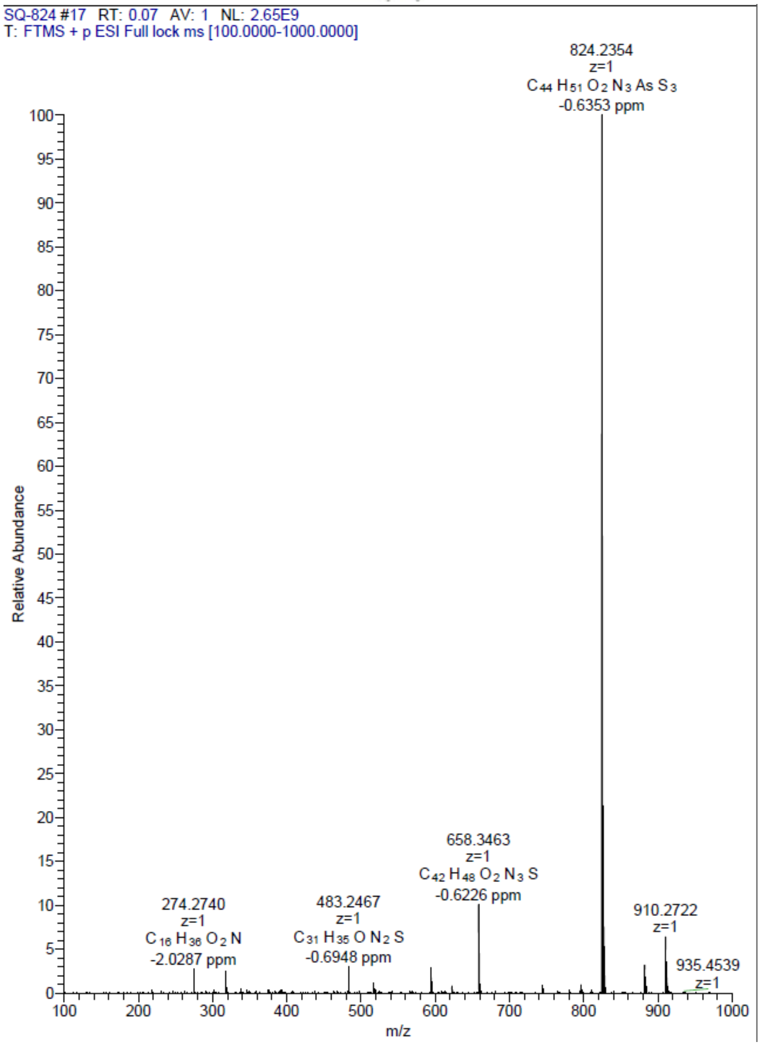

Supplementary Figure 23

^{1}H, ^{13}C NMR and mass spectra (HPLC and High-Resolution Mass Spectra) of MitoESq-635

Supplementary References

1 Koho, S. *et al.* Fourier ring correlation simplifies image restoration in fluorescence microscopy. *Nature Communications* **10**, 3103 (2019).

2 Lam, S. S. *et al.* Directed evolution of APEX2 for electron microscopy and proximity labeling. *Nature Methods* **12**, 51-54 (2015).

3 Wimmer, N., Robinson, J. A., Gopisetty-Venkatta, N., Roberts-Thomson, S. J., Monteith, G. R., Toth, I., *Medicinal Chemistry* **2**, 79-87 (2006).

4 Tatarets, Anatoliy L., *et al. Dyes and Pigments* **64**, 125-134 (2005)

5 Ilina, K. *et al.* Squaraine Dyes: Molecular Design for Different Applications and Remaining Challenges. *Bioconjugate Chemistry* **31**, 194-213 (2020).

6 Wu, I. C. *et al.* Squaraine-based polymer dots with narrow, bright near-infrared fluorescence for biological applications. *Journal of the American Chemical Society* **137**, 173-178 (2015).

7 Yao, D. *et al.* Molecular Engineered Squaraine Nanoprobe for NIR-II/Photoacoustic Imaging and Photothermal Therapy of Metastatic Breast Cancer. *ACS Applied Materials & Interfaces* **12**, 4276−4284 (2020).

8 Griffin, B. A., Adams, S. R. & Tsien, R. Y. Specific covalent labeling of recombinant protein molecules inside live cells. *Science* **281**, 269-272 (1998).

9 Wysocki, L. M. *et al.* Facile and general synthesis of photoactivatable xanthene dyes. *Angewandte Chemie International Edition* **50**, 11206-11209 (2011).

10 Hambrock, A., Löffler‐Walz, C. & Quast, U. Glibenclamide binding to sulphonylurea receptor subtypes: dependence on adenine nucleotides. *British journal of pharmacology* **136**, 995-1004 (2002).

11 Erdmann, R. S. *et al.* Super‐Resolution Imaging of the Golgi in Live Cells with a Bioorthogonal Ceramide Probe. *Angewandte Chemie International Edition* **53**, 10242-10246 (2014).

12 Simmons, D. L., Botting, R. M. & Hla, T. Cyclooxygenase Isozymes: The Biology of Prostaglandin Synthesis and Inhibition. *Pharmacological Reviews* **56**, 387, doi:10.1124/pr.56.3.3 (2004).

13 Velapoldi, R. A. & Tønnesen, H. H. Corrected emission spectra and quantum yields for a series of fluorescent compounds in the visible spectral region. *Journal of fluorescence* **14**, 465-472 (2004).

14 Stephan, T., Roesch, A., Riedel, D. & Jakobs, S. Live-cell STED nanoscopy of mitochondrial cristae. *Scientific Reports* **9**, 12419, doi:10.1038/s41598-019-48838-2 (2019).

15 Wang, C. *et al.* A photostable fluorescent marker for the superresolution live imaging of the dynamic structure of the mitochondrial cristae. *Proceedings of the National Academy of Sciences* **116**, 15817-15822 (2019).

16 Huang, X. *et al.* Fast, long-term, super-resolution imaging with Hessian structured illumination microscopy. *Nature biotechnology* **36**, 451 (2018).

17 Wäldchen, S., Lehmann, J., Klein, T., Van De Linde, S. & Sauer, M. Light-induced cell damage in live-cell super-resolution microscopy. *Scientific reports* **5**, 15348 (2015).

18 Laissue, P. P., Alghamdi, R. A., Tomancak, P., Reynaud, E. G. & Shroff, H. Assessing phototoxicity in live fluorescence imaging. *Nature methods* **14**, 657-661 (2017).
