# Supplementary material for: Mitochondrial dynamics quantitatively revealed by STED nanoscopy with an enhanced squaraine variant probe
Source: Nat Commun. 2020 Jul 24;11:3699. doi: 10.1038/s41467-020-17546-1 (PMC7382495; doi:10.1038/s41467-020-17546-1)
Supplement: Supplementary file 3 — Description of Additional Supplementary Files [file 41467_2020_17546_MOESM3_ESM.pdf]

**Description of Additional Supplementary Files**

File Name: Supplementary Movie 1
Description: Drop1 KO HeLa with long mitochondria also have tendency to become bubbles, but with less speed. Dyed with 100 nM MitoESq-635 and with Prolong live antifade reagent (P36975, Thermo Fisher) for reducing photobleaching. 3s/frame, walking average, scale bar: 1 µm. 200 frames, 10 min. (a) Excitation 633 nm, 5 µW; STED 775 nm, 8.96 mW. (b) Excitation 633nm, 5uW; STED 775nm, 7.84 mW. (c) Excitation 633 nm, 2.5 µW; STED 775 nm, 6.72 mW.

File Name: Supplementary Movie 2
Description: The raw data for the STED images (1024×1024 pixels) in different layers of Figure 2(b).

File Name: Supplementary Movie 3
Description: The raw data for the STED images (1024×1024 pixels) in different layers of Figure 2(c).

File Name: Supplementary Movie 4
Description: The raw data for the STED images of Figure 3(a). Time-lapse STED imaging of the mitochondria.

File Name: Supplementary Movie 5
Description: The raw data for the STED images of Figure 4(a). Time-lapse STED imaging of the mitochondria show typical mitochondria fission processes. Scale bar, 2 µm.

File Name: Supplementary Movie 6
Description: The raw data for the STED images of Figure 4(f). Time-lapse STED imaging of the mitochondria show mitochondria fussion processes. Scale bar, 2 µm.

File Name: Supplementary Movie 7
Description: The raw data for the STED images of Figure 4(h). Time-lapse STED imaging of the mitochondria show mitochondria fussion processes. Scale bar, 1 µm.
