# Supplementary material for: Mitochondrial dynamics quantitatively revealed by STED nanoscopy with an enhanced squaraine variant probe
Source: Nat Commun. 2020 Jul 24;11:3699. doi: 10.1038/s41467-020-17546-1 (PMC7382495; doi:10.1038/s41467-020-17546-1)
Supplement: Supplementary file 11 — Reporting Summary [file 41467_2020_17546_MOESM11_ESM.pdf]

nature research

Corresponding author(s): Peng Xi

Last updated by author(s): Jun 12, 2020

Reporting Summary

Nature Research wishes to improve the reproducibility of the work that we publish. This form provides structure for consistency and transparency in reporting. For further information on Nature Research policies, see Authors & Referees and the Editorial Policy Checklist.

Statistics

For all statistical analyses, confirm that the following items are present in the figure legend, table legend, main text, or Methods section.

n/a	Confirmed	
☐	☒	The exact sample size (*n*) for each experimental group/condition, given as a discrete number and unit of measurement
☒	☐	A statement on whether measurements were taken from distinct samples or whether the same sample was measured repeatedly
☒	☐	The statistical test(s) used AND whether they are one- or two-sided<br>*Only common tests should be described solely by name; describe more complex techniques in the Methods section.*
☒	☐	A description of all covariates tested
☒	☐	A description of any assumptions or corrections, such as tests of normality and adjustment for multiple comparisons
☐	☒	A full description of the statistical parameters including central tendency (e.g. means) or other basic estimates (e.g. regression coefficient) AND variation (e.g. standard deviation) or associated estimates of uncertainty (e.g. confidence intervals)
☒	☐	For null hypothesis testing, the test statistic (e.g. *F*, *t*, *r*) with confidence intervals, effect sizes, degrees of freedom and *P* value noted<br>*Give P values as exact values whenever suitable.*
☒	☐	For Bayesian analysis, information on the choice of priors and Markov chain Monte Carlo settings
☒	☐	For hierarchical and complex designs, identification of the appropriate level for tests and full reporting of outcomes
☒	☐	Estimates of effect sizes (e.g. Cohen's *d*, Pearson's *r*), indicating how they were calculated

Our web collection on statistics for biologists contains articles on many of the points above.

Software and code

Policy information about availability of computer code

Data collection	Leica SP2 and SP8 for imaging
Data analysis	(1)Open source (MIPLIB): https://github.com/sakoho81/miplib(ref. 26); for FRC.<br>(2) Fiji based on ImageJ 2.0.0-rc-69/1.52p; for detection of width of mitochondria, and fluorescence intensity.<br>(3) OriginPro 9.1 64 bit; for box plot and error bar charts.<br>(4) Origin 2018; for Gaussian Cumulative Peak Fit.

For manuscripts utilizing custom algorithms or software that are central to the research but not yet described in published literature, software must be made available to editors/reviewers. We strongly encourage code deposition in a community repository (e.g. GitHub). See the Nature Research guidelines for submitting code & software for further information.

Data

Policy information about availability of data

All manuscripts must include a data availability statement. This statement should provide the following information, where applicable:

- Accession codes, unique identifiers, or web links for publicly available datasets
- A list of figures that have associated raw data
- A description of any restrictions on data availability

The data that support the findings of this study are available through "figshare.com" with the identifier(s) "https://doi.org/10.6084/m9.figshare.12252842"34. The data that support the findings of this study are available from the corresponding author upon reasonable request. The MitoESq-635 dye can be obtained from the author (Z.Y.) upon reasonable request.

Field-specific reporting

Please select the one below that is the best fit for your research. If you are not sure, read the appropriate sections before making your selection.

☒ Life sciences ☐ Behavioural & social sciences ☐ Ecological, evolutionary & environmental sciences

For a reference copy of the document with all sections, see nature.com/documents/nr-reporting-summary-flat.pdf

Life sciences study design

All studies must disclose on these points even when the disclosure is negative.

Sample size	n=6 were choose for box plot and error bars in main text, n=5 were choose for error bars in Supplementary Figures, using 5-6 points can exclude extreme values.
Data exclusions	No data were excluded from analyses.
Replication	Figures 1d and 1e; 2a-d; 3a, b; SI Figure 4, 5, 7d-e, 8a-b, 10a, 11a,12 were repeated for 2 times with similar results. Figures 4a-b, f, h, i; SI Figure 1-3, 13 were repeated for 3 times with similar results.
Randomization	Samples are allocated randomly.
Blinding	Blinding was not relevant to this study, because our experimental subjects are cells rather than patients.

Reporting for specific materials, systems and methods

We require information from authors about some types of materials, experimental systems and methods used in many studies. Here, indicate whether each material, system or method listed is relevant to your study. If you are not sure if a list item applies to your research, read the appropriate section before selecting a response.

Materials & experimental systems

n/a	Involved in the study	
☒	☐	Antibodies
☐	☒	Eukaryotic cell lines
☒	☐	Palaeontology
☒	☐	Animals and other organisms
☒	☐	Human research participants
☒	☐	Clinical data

Methods

n/a	Involved in the study	
☒	☐	ChIP-seq
☒	☐	Flow cytometry
☒	☐	MRI-based neuroimaging

Eukaryotic cell lines

Policy information about cell lines

Cell line source(s)	Hela cell, MCF 7, U2OS, SH-SY5Y, SKOV, RAW 264.7. Some Hela cells are purchased from American Type Culture Collection (ATCC). Other Cell lines were purchased from the Procell (Wuhan, China)
Authentication	Cell lines were authenticated by the commercial vendors.
Mycoplasma contamination	The cell lines were not tested for mycoplasma contamination.
Commonly misidentified lines (See ICLAC register)	No commonly misidentified lines.
